# Total Synthesis of Novel Skeleton Flavan-Alkaloids

**DOI:** 10.3390/molecules25194491

**Published:** 2020-09-30

**Authors:** Jing Zhen, James E. Simon, Qingli Wu

**Affiliations:** 1 Department of Medicinal Chemistry, Ernest Mario School of Pharmacy, Rutgers University, 160 Frelinghuysen Road, Piscataway, NJ 08854, USA; zhenjing89@gmail.com; 2 The New Use Agriculture and Natural Plant Products Program, Department of Plant Biology, Rutgers University, 59 Dudley Road, New Brunswick, NJ 08901, USA

**Keywords:** *Combretum micranthum*, kinkeloids A, kinkeloids B

## Abstract

The first total synthesis of novel skeleton natural compounds kinkeloids A and B, a group of newly discovered flavan alkaloids isolated from the African plant *Combretum micranthum*, are described in this study. The key and final step are achieved by Mannich reaction, through which the piperidine moiety couples to the flavan moiety. The identities of synthesized kinkeloids were further confirmed through a comparison with the ones in the plant leaves extract using LC/MS.

## 1. Introduction

**Figure 1:**
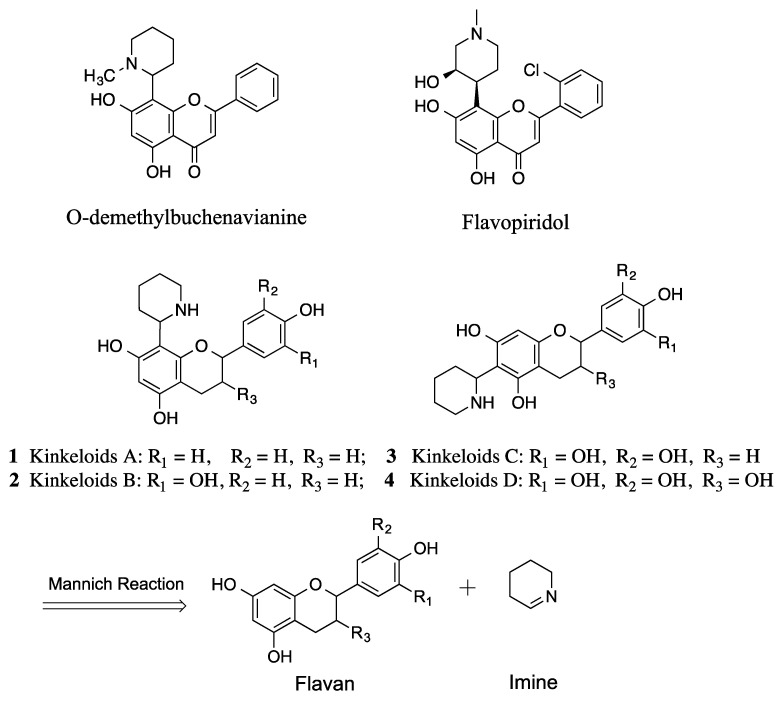
Kinkeloids and retrosynthetic analysis.

Kinkeloids are a series of newly discovered natural compounds with a novel skeleton from the leaves of the shrub plant *Combretum micranthum*, which is indigenous to Senegal and West Africa and commonly called kinkeliba. The leaves of kinkeliba are widely consumed as an herbal tea in West Africa and for the treatment of gastrointestinal problems, colic and vomiting [1,2]. The beverage brewed from the dried leaves is generally used as a panacea for possible weight loss and has shown antihyperglycemic activity in vivo [3]. Kinkeloids belong to the family of flavonoid alkaloids, which are chemically interesting as the skeletons can serve as a platform for targeted drug design due to their amphoteric structure with phenolic acids and basic nitrogenous moieties. The flavonoid alkaloids family has been shown to exhibit a wide range of biological activities including antiviral, anticancer, and anti-inflammatory activities [4]. For example, flavone alkaloids *O*-demethylbuchenavianine from *Buchenavia capitata* have shown HIV-inhibitory activity with an EC_50_ of 0.26 µM [5]. Flavopiridol derived from natural chrome alkaloid rohitukine that was extracted from *Dysoxylum binectariferum* can inhibit the activity of cyclin-dependent kinases (CDKs), and is being developed in clinical trial for cancer treatment [6] (Figure 1).

Kinkeloids were first discovered by our group [1]. Their structures are mainly made up of a flavan moiety with a piperidine ring attached at either the 6 or 8 position. Kinkeloids A, B, C, and D differ from each other only by the number of hydroxyl groups attached, resulting in consecutive mass unit difference in mass spectra (Figure 1). Since kinkeloids have been identified as the major component contained in *C. micranthum*, which has traditionally been used in herbal tea, it was of interest to explore some of their biological activities. Due to the close physiochemical properties between theses kinkeloids in the plant extract, direct separation and accumulation using chromatography is very challenging and time-consuming, especially for large-scale production during pharmacological research.

## 2. Results and Discussion

Here, we report on two synthetic methods that would be used for the preparation of kinkeloids. Both synthetic routes are based on the Mannich reaction, which links the flavan moiety and the piperidine moiety (Figure 1). To the best of our knowledge, only flavone and flavanone classes have been reported from the family of flavonoids in the synthesis of their corresponding flavonoid alkaloids through a reaction with cyclic imine [7,8,9]. The chemical properties between different classes of flavonoids vary greatly and affect reaction results. In our study, the Mannich reaction between a flavan molecule and cyclic imine was explored for the first time. As reported, the other flavonoid alkaloids usually have low solubility in alcohol and even in DMSO, and the 8-substitued groups generally are less soluble than the 6-substitued ones. In contrast with other flavonoid alkaloids, the synthesized flavan alkaloids are very soluble in alcohol, including methanol and ethanol. Therefore, the solvent selected to perform the flavan Mannich reaction was 100% methanol. Due to the comparatively higher flexibility of flavan molecules, the substitution on the 6-,8-positions had a lack of selectivity, even when different solvent systems were tried including MeOH, EtOH, H_2_O/MeOH, H_2_O/THF. Yet, this substitution selectivity was observed and reported in flavonone and flavone groups, indicating that carbonyl groups at position 4 can impact the nucleophilic activities of positions 6 and 8 [9,10].

**Scheme 1:**
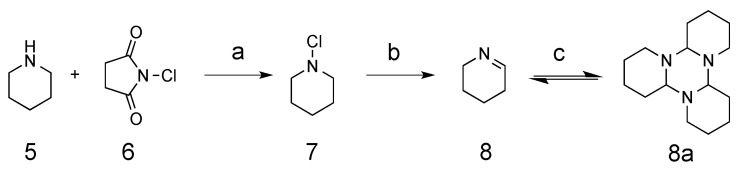
The synthesis of Δ^1^-piperidein intermediate. Reaction conditions and reagents: (**a**) ether, RT; (**b**) 20% KOH, ethanol, 0 °C to RT; (**c**) 80% acetone, −20 °C.

The synthesis of cyclic imine started from commercially available compound piperidine, which was converted to *N*-chloropiperidine **7** through the addition of *N*-chlorosuccinimide (NCS). Treatment of the *N*-chloropiperidine formed with potassium hydroxide in ethanol provided the corresponding synthetic intermediate Δ^1^-piperidein **8**. In this step, low yields were observed when excessive heating was used for solvent removal because of the low boiling point of compound **8**. Δ^1^-piperidein was then crystalized out in its trimeric form as tripiperidein **9** in cold acetone (Scheme 1) [11].

**Scheme 2:**
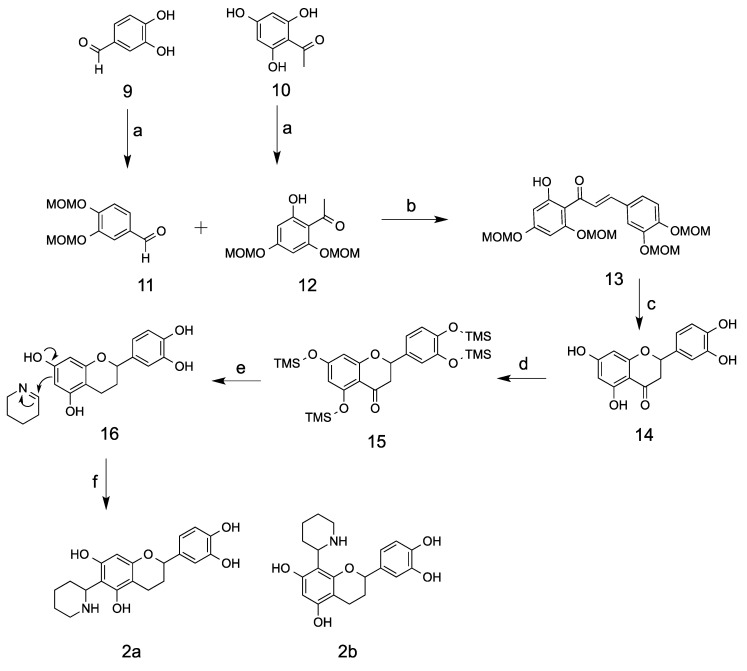
Route one for the synthesis of kinkeloids B. Reaction conditions and reagents: (**a**) DIPEA, MOMCl, DMAP, CH_2_Cl_2_, 0 °C to RT (88% for **11**, 74% for **12**); (**b**) 40% KOH, ethanol, 0 °C to RT (62%); (**c**) 10% HCl, methanol, reflux (60%); (**d**) TMSCl, HMDS, pyridine (**e**) LiBH_4_, NaBH_3_CN, THF, 10% HCl, RT (65%); (**f**) Δ^1^-piperideine, methanol, 70 °C (20%).

**Scheme 3:**
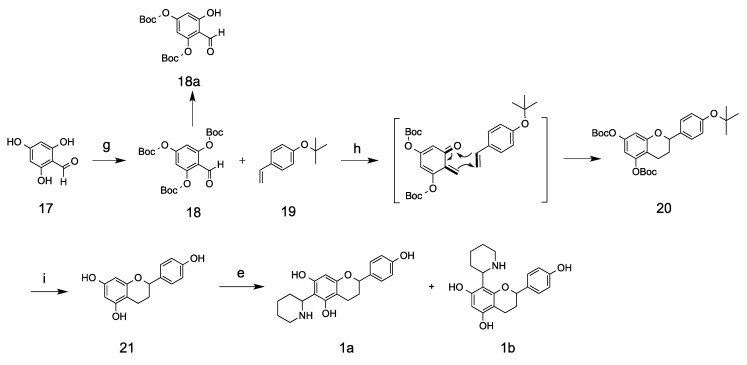
Route two for the synthesis of kinkeloids A. Reaction conditions and reagents: (**g**) Boc_2_O, DIPEA, DMAP, CH_2_Cl_2_, RT, overnight (46.5%); (**h**) MgBr_2_·OEt_2_, LiAlH_4_, ether, −78 °C to RT; (**i**) ZnBr2, CH_2_Cl_2_, (9.7%); (**e**) Δ^1^-piperideines, methanol, 70 °C (25%).

The two synthetic methods presented here differ from each other in the synthesis of flavan moiety [12,13]. Synthetic method I was applied for the synthesis of the kinkeloid B series (Scheme 2), while method II was used for the A series (Scheme 3). Synthetic method I started from the commercially available compounds 3,4-dihydroxybenzaldehyde and 2,4,6-trihydroxyacetophone, which were protected with MOM groups to provide compounds **11** and **12**. MOM groups are an ideal selection due to its stability in the basic condition and its facile removal in an acidic condition. The ethanol mixture of **11** and **12** was then basified with 40% KOH solution in an ice bath and gradually warmed up to room temperature to provide a 62% yield of fully protected chalcone **13** [14]. De-protection of MOM groups using 10% HCl in methanol under an elevated temperature of 50 °C yielded exclusively the compound eriodictyol **14**, which was cyclized from de-protected chalcone intermediate. The phenolic acid groups were then fully protected with trimethylsilyl (TMS) groups before the step of reductive de-oxygenation using mild reducing reagent NaBH_3_CN. NaBH_3_CN, as a reducing agent, has remarkable stability in acidic environments, whose hydrolysis rate is 10^−8^ that of sodium borohydride [15]. A wide range of acids including BF_3_·OEt_2_, ZnCl_2_, and concentrated HCl, were compared in the reaction and it was found that 10% HCl delivered the best conversion (60% yield) without opening the A ring at the 2 position [16,17]. The TMS protecting groups were also removed in the reaction by hydrolysis. Flavan **16** reacted with Δ^1^-piperidein and yielded the kinkeloid B series with a 20% yield. Low yields may have been due to the formation of a byproduct, 6,8-di-piperidine-substituted flavan, and a low conversion percentage.

Synthetic method II involved the application of *o*-quinone methide and an inverse electron-demand Diels–Alder reaction. The starting material 2,4,6-trihydroxybenzaldehyde was reacted with di-*tert*-butyl dicarbonate to form the ortho *o*-boc compound **18**, which is thermally unstable on silica column and can decompose to the di-boc-substituted compound **18a** at room temperature. The use of a cold solvent system (ethyl acetate/hexane) from a −20 °C freezer for chromatographic separation prevents its decomposition and keeps its tri-substituted status. Compound **18** was then used to generate *o*-quinone methides in combination with magnesium bromide and lithium aluminum hydride [18]. The generation of *o*-quinone methides through Grignard regents requires extreme dryness. Subsequently, the 1,4-conjugate addition of *o*-quinone methide with electron-rich olefins through an inverse Diels–Alder reaction yielded the protected flavan products. A low yield was observed in our experiment, which we attributed to the product’s insufficient dryness during the process. Higher yields of aldehydes, alkenes, and other materials have been reported when kept extremely dry, using sodium through Kugelrohr distillation [19], and this inverse Diels–Alder reaction has previously been applied to the synthesis of modified flavonoid molecules [19]. The *t*-butyl and Boc groups were subsequently removed using ZnBr_2_ at room temperature, and 4′,5,7-trihydroxyl flavan was achieved [20]. Kinkeloids A was synthesized through a reaction between flavan **21** and Δ^1^-piperidein, with a yield of 25%. Compared with the kinkeloid B series, the A series compounds are comparatively less stable and could be easily oxidized to purple compound when exposed to ambient air within just a few hours. This phenomenon correlates very well with the relative amount found in the leaf extract, where the kinkeloid B series is very abundant, while the kinkeloid A series is at trace levels. Both synthesized kinkeloids A and B were further positively confirmed through a comparison with compounds contained in kinkeliba leaf extract on LC/UV/MS, and it was found that the synthesized compounds shared identical UV profiles, mass spectrums and retention times with the ones extracted from the kinkeliba leaves.

In summary, this study presents the first synthesis of kinkeloid molecules, which are a newly discovered flavonoid alkaloids. The synthetic approach in the preparation of such flavan alkaloids allows for the procurement of large quantities that are needed for in vivo efficacy and toxicity studies. Such synthetic derivatives also contribute to the in vivo disposition, metabolism and pharmacokinetic studies through the instillation of stable radioisotopes.

## 3. Materials and Methods

### 3.1. General Experimental Procedures

^1^H and ^13^C-NMR spectra (Supplementary Materials) were performed on a Bruker Avance 400 MHz spectrometer (Billerica, MA, USA). Analytical LC-MS was performed on a Hewlett-Packard Agilent 1100 series HPLC-MSD (Agilent Technologies, Waldbronn, Germany) equipped with a quaternary pump system, a degasser, an auto-sampler, a DAD detector, an MSD trap with an electrospray ion source (ESI). Column chromatography was performed using silica gel (230–400 mesh; Selecto Scientific, Suwanee, GA, USA) and Sephadex LH-20 (25–100 μ, Sigma-Aldrich, St. Louis, MO, USA). All synthetic materials including the solvents were purchased from Sigma Aldrich.

### 3.2. Δ^1^-Piperidein

To a suspension solution of *N*-Chlorosuccinimide **6** (5.9 g, 45.0 mmol) in ether (50 mL), piperidine **5** (2.5 mL, 25.25 mmol) was added drop-wise over 30 min. After being stirred for 3 h at room temperature, the suspension was filtered and washed with ether (20 mL). The combined organic solvent was washed with water (20 mL), brine (20 mL), then dried using anhydrous sodium sulfate. The solvent was removed under reduced pressure, yielding intermediate *N*-chloropiperidine **7**, which was added drop-wise into a solution of potassium hydroxide (3 g) in ethanol (15 mL) followed by stirring for another 5 h at room temperature. After the reaction, diethyl ether (25 mL) was added to the solution, which was washed with water and brine, then dried using anhydrous sodium sulfate. Solvent ether was then removed under reduced pressure without heating, which yielded the product Δ^1^-piperidein (1.30 g, 62%). A low yield was observed if the solvent was removed under heating. Δ^1^-piperidein was crystalized as tripiperidein in cold acetone at −20 °C. ^1^H-NMR (400 MHz CDCl_3_), a mixture of monomeric and trimeric forms. Monomeric form (some peaks overlapped with the trimeric form): δ 7.77–7.86 (m, 1H, N=C-H), 3.52–3.60 (m, 2H, -CH_2_-), 2.33 (m, 2H, -CH_2_-), 2.13 (m, 2H, -CH_2_-). Trimeric form: 3.07–3.11 (m, 1H, -CH-), 2.76–2.78 (m, 1H, -CH_2_-), 1.95–1.99 (m, 1H, -CH_2_-), 1.65–1.69 (m, 3H, 2 × -CH_2_-), 1.52–1.54 (m, 2H, -CH_2_-), 1.12–1.29 (m, 2H, -CH_2_-). ^13^C-NMR (100 MHz CDCl_3_) monomeric form (some peaks overlapped with the trimeric form): δ 163.2 (CH, -N=CH-), 49.35 (CH_2_, -CH_2_-N=), 28.85 (CH_2_, -CH_2_-CH=), 18.80 (CH_2_, -CH_2_-); trimeric forms: 82.05 (CH, -N-CH (CH_2_)-N-); 46.48 (CH2, -CH_2_-N-), 29.27 (CH_2_, -CH_2_-), 25.87 (CH_2_, -CH_2_-), 22.41 (CH_2_, -CH_2_-).

### 3.3. 3,4-bis(methoxymethoxy)benzaldehyde (**11**)

To an ice-cooled suspension of 3,4-dihydroxybenzaldehyde (500 mg, 3.62 mmol) in 10 mL of dichloromethane, *N,N*-diisopropylethylamine (6.0 mL) and DMAP (5 crystals) were added. Then, MOMCl (chloromethyl methyl ether, 642 mg, 7.96 mmol) was added drop-wise at 0 °C, and the reaction was gradually warmed up to room temperature and stirred overnight. After the disappearance of the starting material on TLC, the reaction was poured into water (10 mL) and extracted with organic solvent dichloromethane (2 × 20 mL). The combined organic phase was washed with water, brine and dried over anhydrous sodium sulfate. The solvent was then removed under reduced pressure and the residue was further purified on silica column using hexane/ethyl acetate (9:1) to yield compound **11** as a white solid (720 mg, 88%). ^1^H-NMR (400 MHz CDCl_3_) δ 3.51 (3H, s, -OCH_3_), 3.52 (3H, s, -OCH_3_), 5.28 (2H, s, -OCH_2_O-), 5.32 (2H, s, -OCH_2_O-), 7.27 (1H, d, *J* = 8.4 Hz, H-5′), 7.50 (1H, dd, *J* = 8.4 and 1.9, H-6′), 7.67 (1H, d, *J* = 1.9 Hz, H-2′), 9.86 (1H, s, -CHO); ^13^C-NMR (100 MHz CDCl_3_) 56.5 (CH_3_, -OCH_3_), 56.6 (CH_3_, -OCH_3_), 95.1 (CH_2_, -OCH_2_O-), 95.5 (CH_2_, -OCH_2_O-), 115.6 (CH, C-5′), 116.1 (CH, C-2′), 126.4 (CH, C-6′), 131.3 (C, C-1′), 147.6 (C, C-3′), 152.8 (C, C-4′), 190.9 (C, CHO).

### 3.4. 1-(2-hydroxy-4,6-bis(methoxymethoxy)phenyl)ethan-1-one (**12**)

2,4,6-trihydroxyacetophone (500 mg, 2.97 mmol) was suspended in ice-old dichloromethane (10 mL) followed by the addition of *N,N*-diisopropylethylamine (6.0 mL) and DMAP (5 crystals). After around 10 min, MOMCl (515 mg, 6.40 mmol) was added drop-wise and the mixture was stirred overnight. After the disappearance of starting material shown on TLC, the mixture was poured into water (10 mL), and extracted with dichloromethane (2 × 20 mL) two times. The organic phase was washed with water (10 mL) and brine (10 mL), and dried using anhydrous sodium sulfate. The solvent was then removed under reduced pressure and the residue was purified on silica gel column using hexane/ethyl acetate (9:1) to yield compound **12** (560 mg, 73.4%) as a colorless oil that was crystalized as a white solid. ^1^H-NMR (400 MHz CDCl_3_) δ 2.66 (s, 3H, -COCH_3_), 3.47 (s, 3H, -OCH_3_), 3.51 (3H, s, -OCH_3_), 5.17 (2H, s, -OCH_2_O-), 5.25 (2H, s, -OCH_2_O-), 6.24 (1H, d, *J* = 2.3 Hz, Ar-H), 6.27 (1H, d, *J* = 2.3 Hz, Ar-H); ^13^C-NMR (100 MHz CDCl_3_) δ 33.1 (CH_3_, -COCH_3_), 56.6 (CH_3_, -OCH_3_), 56.9 (CH_3_, -OCH_3_), 94.2 (CH_2_, 2 × -OCH_2_O-), 94.7 (CH, C-5′), 97.4 (CH, C-3′), 107.1 (C, C-1′), 160.5 (C, C-6′), 163.6 (C, C-4′), 167.0 (C, C-2′), 203.4 (C=O, C-1).

### 3.5. 3-(3,4-bis(methoxymethoxy)phenyl)-1-(2-hydroxy-4,6-bis(methoxymethoxy)phenyl)prop-2-en-1-one (**13**)

Compounds **11** (160 mg, 0.71 mmol) and **12** (150 mg, 0.59 mmol) were co-suspended in an ice-cold solution of ethanol (5 mL) followed by the addition of 40% KOH solution (1 mL). The mixture was gradually warmed to room temperature and stirred overnight. After that, the reaction was poured into water (10 mL) and extracted with dichloromethane (3 × 20 mL). The combined organic phase was washed with water (10 mL) and brine (10 mL), and dried using anhydrous Na_2_SO_4_. The solvent was then purified on silica column using hexane/ethyl acetate (5:1) to obtain compound **13** (170 mg, 62.0%) as a yellow solid. ^1^H-NMR (400 MHz CDCl_3_) δ 3.48 (3H, s, -OCH_3_), 3.52 (3H, s, -OCH_3_), 3.53 (3H, s, -OCH_3_), 3.54 (3H, s, -OCH_3_), 5.18 (2H, s, -OCH_2_O-), 5.27 (2H, s, -OCH_2_O-), 5.28 (2H, s, -OCH_2_O-), 5.30 (2H, s, -OCH_2_O-), 6.27 (1H, d, *J* = 2.3 Hz, H-3′), 6.31 (1H, d, *J* = 2.3 Hz, H-5′), 7.17–7.22 (2H, m, H-5, 6), 7.51 (1H, d, *J* = 1.7 Hz, H-2). 7.74 (1H, d, *J* = 15.5, H-α), 7.86 (1H, d, *J* = 15.5, H-β), 13.92 (1H, s, -OH); ^13^C-NMR (100 MHz CDCl_3_) δ 56.4 (CH_3_, -OCH_3_), 56.5 (CH_3_, -OCH_3_), 56.6 (CH_3_, -OCH_3_), 57.0 (CH_3_, -OCH_3_), 94.2 (CH_2_, -OCH_2_O-), 94.9 (CH_2_, -OCH_2_O-), 95.2 (CH_2_, -OCH_2_O-), 95.3 (CH_2_, -OCH_2_O-), 95.7 (CH, C-5′), 97.7 (CH, C-3′), 107.7 (C, C-1′), 115.5 (CH, C-2), 116.4 (CH, C-5), 124.3 (CH, C-6), 126.1 (CH, C-α), 130.1 (C, C-1), 142.5 (CH, C-β), 147.7 (C, C-4), 149.3 (C, C-3), 160.1 (C, C-2′), 163.5 (C, C-6′), 167.5 (C, C-4′), 192.9 (C, C=O).

### 3.6. 2-(3,4-dihydroxyphenyl)-5,7-dihydroxychroman-4-one (**14**)

Compound **13** (70 mg, 0.15 mmol) was suspended in methanol (5 mL) followed by the addition of 37% HCl solution (1 mL). The mixture was heated up and refluxed for 5 h. After the reaction, the solution was cooled down and poured into water, then extracted with EtOAc (3 × 10 mL). The combined organic phases were washed with water and brine, then dried using anhydrous Na_2_SO_4_. Then solvent was removed under reduced pressure. The residue was dissolved in methanol (0.5 mL) and purified over Sephadex LH-20 column using 100% methanol to yield compound **14** (30.5 mg, 71%) as a white solid. This compound could also be purified on silica gel column using solvent hexane/ethyl acetate (4:1). ^1^H-NMR (400 MHz CDCl_3_) δ 2.67 (1H, dd, *J* = 3.0 Hz and 17.1 Hz, H-3), 3.19 (1H, dd, *J* = 17.1 and 12.5 Hz, H-3), 5.37 (1H, dd, *J* = 12.5 and 3.0 Hz, H-2), 5.87 and 5.88 (2H, d, H-6 and 8), 6.74 (2H, s, H-5′ and 6′), 6.87 (1H, s, H-2); ^13^C-NMR (100 MHz CDCl_3_) δ 42.1 (CH, C-3), 78.4 (CH, C-2), 94.9 (CH, C-6), 95.7 (CH, C-8), 101.8 (C, C-10), 114.3 (CH, C-2′), 115.3 (CH, C-5′), 117.9 (CH, C-6′), 129.4 (C, C-1′), 145.2 (C, C-4′), 145.7 (C, C-3′),162.9 (C, C-5), 163.4 (C, C-9), 166.6 (C, C-7), 196.3 (C, C-4).

### 3.7. 2-(3,4-dihydroxyphenyl)chromane-5,7-diol (**16**)

To a solution of compound **14** (288 mg, 1.0 mmol) in dry pyridine (1 mL) was added hexamethyldisilazane (1 mL) and trimethylchlorosilane (0.6 mL). The mixture was then further stirred at room temperature for 1 h. The volatile constituents were distilled out under reduced pressure; the residue was suspended in toluene and the precipitate was then filtered out. Solvent toluene solution was then distilled out under reduced pressure and the residue was dissolved in THF (2.5 mL) followed by the addition of LiBH_4_ (11 mg, 0.5 mmol). The mixture was further stirred at room temperature for half an hour. Next, 0.5 mg of methyl orange and sodium cyanoborohydride (63 mg, 1.0 mmol) were added into the solution. The mixture was slowly titrated using hydrochloric acid solution (1 N) with the release of hydrogen until the color of the solution turned red. The color of the solution was controlled in red by the slow, continuous addition of hydrochloric acid. After approximately 2 h, an extra amount of hydrochloric acid (1 to 1.5 mL) was added to make the color permanent, and the reaction was stirred overnight at room temperature. After this, the solvent THF was distilled out under reduced pressure and the residue was extracted with ethyl acetate and washed with 1 N hydrochloric acid solution, distilled water, and brine. The organic solution was dried using anhydrous sodium sulfate and purified on silica column using 10–20% ethyl acetate in hexane as eluent in gradient with a 65% yield. ^1^H-NMR (400 MHz, MeOD-*d*_4_) δ 1.88 (1H, m, H-3), 2.08 (1H, m, H-3), 2.61 (2H, m, H-4), 4.77 (1H, dd, H-2), 5.84 (1H, d, *J* = 2.3 Hz, H-8), 5.90 (1H, d, *J* = 2.3 Hz, H-6), 6.67 (2H, m, H-5′,6′), 6.85 (1H, d, *J* = 1.84, H-2′); ^13^C-NMR (100 MHz, MeOD-*d*_4_) δ 20.35 (C-4), 30.89 (C-3), 78.848 (C-2), 96.00 (C-8), 96.08 (C-6) 102.6 (C-10), 114.47 (C-5′), 116.17 (C-2′), 118.83 (C-6′), 135.14 (C-1′), 145.93 (C-3′), 146.24 (C-4′), 157.31, 157.49, 157.97 (C-5,7,9).

### 3.8. Kinkeloids B

The synthesized flavan compound **16** (40 mg, 0.15 mmol) was dissolved in methanol (4 mL) followed by the addition of Δ^1^-piperideines **8** (12.1 mg, 0.15 mmol). The mixture was stirred at 70 °C under nitrogen atmosphere for 4 h. After the reaction, the solvent was removed under reduced pressure and the residue was purified on Sephadex column LH-20 using methanol to produce a mixture of compound **2a** and **2b** (10 mg, 20%, the low yield was due to the formation of di-piperidine-substituted products). Compound **2a**: ^1^H-NMR (400 MHz, DMSO-*d*_6_): δ 1.30–1.72 (6H, m, H-4″, 5″,6″), 1.72–1.88 (1H, m, H-3), 2.00 (1H, m, H-3), 2.48 (2H, m, H-4) 2.60 (1H, m, H-3″), 3.08 (1H, m, H-3″), 4.10 (1H, m, H-1″), 4.73 (1H, m, H-2), 5.78 (1H, S, H-8), 6.61–6.72 (2H, m, H-5′,6′), 6.76 (1H, d, *J* = 1.92, H-2′); ^13^C-NMR (100 MHz, DMSO-*d*_6_) δ 19.0 (C-4), 24.0, 24.5 (C-4″,5″), 29.0 (C-3), 30.3 (C-6″), 45.7 (C-3′’) 52.8 (C-1″), 76.4 (C-2), 93.9 (C-8), 101.4 (C-10), 107.1 (C-6), 113.7 (C-2′), 115.3 (C-5′), 117.1 (C-6′), 132.7 (C-1′), 144.8, 145.1 (C-3′,4′) 153.2, 154.6, 155.9 (C-5,7,9). Compound **2b**: ^1^H-NMR (400 MHz DMSO-*d*_6_): δ 1.30–1.72 (6H, m, H-4″,5″,6″), 1.72–1.88 (1H, m, H-3), 2.00 (1H, m, H-3), 2.50 (2H, m, H-4) 2.70 (1H, m, H-3″), 3.16 (1H, m, H-3″), 4.20 (1H, m, N-H), 4.86 (1H, m, H-2), 5.97 (1H, S, H-6), 6.61–6.8 (3H, m, H-5′,6′,2′); ^13^C-NMR (100 MHz, DMSO-*d*_6_) δ 18.7 (C-4), 23.3 23.5 (C-4″,5″), 28.6 (C-3), 29.6 (C-6″), 45.5 (C-3″) 52.5 (C-1″), 76.4 (C-2), 95.3 (C-6), 100.2 (C-10), 104.0 (C-8), 113.4 (C-2′), 115.4 (C-5′), 116.5 (C-6′), 132.7 (C-1′), 144.8, 145.2 (C-3′,4′) 152.7, 155.3, 155.5 (C-5,7,9).

### 3.9. Tri-tert-butyl (2-formylbenzene-1, 3, 5-triyl) tricarbonate (**18**)

2,4,6-trihydroxybenzaldehyde (1.0 g, 6.50 mmol) was placed in an oven-dried flask filled with nitrogen, followed by dissolution using solvent CH_2_Cl_2_. Next, *N,N*-diisopropylethylamine (DIPEA) (0.7 mL), catalyst DMAP (four crystals), and Boc_2_O (5 mL, 21.8 mmol) were added, and the mixture was stirred at room temperature overnight. The reaction was stopped using excessive 1M NH_4_Cl and was extracted by dichloromethane (2 × 20 mL). The combined organic solvent was washed with water (10 mL) and brine (10 mL), then dried using anhydrous MgSO_4_. The solution was filtered and dried under reduced pressure. The residue was purified on silica column using refrigerated/cold hexane/ethyl acetate (95:5), yielding compound **18** (1.37 g, 46.5%) as a colorless glue. Cold solvents are necessary for purification due to the instability of tri-Boc protected material, which can decompose to di-Boc protected side product (**18a**) at an elevated temperature. ^1^H-NMR (400 MHz, CDCl_3_) δ 1.55 (9H, s, 3 × -CH_3_), 1.56 (18H, s, 6 × -CH_3_), 7.10 (2H, s, 2 × Ar-H), 10.20 (1H, s, -CHO).

### 3.10. 2-(4-hydroxyphenyl)chromane-5,7-diol (**21**)

The synthesized intermediate compound **18** (100 mg, 0.22 mmol) was dissolved in anhydrous ether and added to a flask containing 1-tert-butoxy-4-vinylbenzene (4 mL, 21 mmol) and magnesium bromide ethyl etherate MgBr_2_·OEt_2_ (58 mg, 0.22 mmol) at −78 °C with the protection of argon. The mixture was added to LiAlH_4_ (9.0 mg, 0.24 mmol) solution in anhydrous ether through a cannula. The reaction was gradually warmed to room temperature and stirred for another 3 h at room temperature. The reaction was quenched using 1M NaHCO_3_ and extracted using ether (3 × 10 mL). The combined organic phase was washed with water (5 mL) and brine (5 mL), then dried using anhydrous Na_2_SO_4_. The solvent was removed and the residue was re-dissolved in the DCM (5 mL) followed by the addition of ZnBr_2_ (250 mg, 2.2 mmol). The mixture was stirred at room temperature overnight. The reaction was quenched with 1M HCl solution followed by extraction with EtOAc (3 × 10 mL). The combined organic phase was dried under reduced pressure and purified on silica column with hexane/ethyl acetate (3:1) to yield compound **16** (5.5 mg, 9.7%) as a white solid. Due to the reaction’s extreme water sensitivity, a low yield was observed. ^1^H-NMR (400 MHz, MeOD-*d*_4_) δ 7.22 (2H, d, *J* = 8.6 Hz, H-3′,5′), 6.77 (2H, d, *J* = 8.6 Hz, H-2′,6′), 5.90 (1H, d, *J* = 2.3, H-6), 5.83 (1H, d, *J* = 2.3, H-8), 4.82 (1H, br, d, H-2), 2.54–2.71 (2H, m, H-4), 1.87–2.13 (2H, m, H-3). ^13^C-NMR (100 MHz, MeOD-*d*_4_) δ 158.09, 157.99, 157.49, 157.33 (C-5,7,9,4′), 134.34 (C-1′), 128.47 (C-3′,5′), 116.05 (C-2′,6′), 96.02 (C-6), 95.94 (C-8), 78.84 (C-2), 30.88 (C-3), 20.43 (C-4).

### 3.11. Kinkeloids A

The synthesized flavan compound **21** (200 mg, 0.78 mmol) was dissolved in methanol followed by the addition of Δ^1^-piperideines (77 mg, 0.93 mmol). The mixture was stirred at 70 °C under nitrogen atmosphere for 5 h. After the reaction, the solvent was removed under reduced pressure and the residue was purified on silica column using methanol/dichloromethane (15:85) to yield a mixture of compound **1a** and **1b**. Further purification on silica gel with methanol/dichloromethae (1:5) and Sephadex column LH-20 with methanol yielded compound **1a** and **1b** as a white solid (25%). It was observed that compounds **1a** and **1b** would be oxidized to purple compounds after a lengthy exposure to the air. Compound **1a**: ^1^H-NMR (400 MHz, DMSO-*d*_6_) δ 1.60 (2H, m, H-5″ and H-4″), 1.88 (4H, m, H-3,4″,5″,6″), 2.09 (2H m, H-3,6″), 2.62 (2H, m, H-4), 2.90 (1H, m, H-3″), 3.30 (1H, d, H-3′’), 4.42 (1H, m, H-1″), 4.88 (1H, m, H-2), 6.03 (1H, S, H-8), 6.80 (2H, d, H-2′,6′), 7.20 (2H, d, H-3′,5′); ^13^C-NMR (400 MHz, DMSO-*d*_6_) δ 19.58 (C-4), 21.8, 22.75 (C-4″,5″) 28.56 (C-6″), 28.54 (C-3), 45.40 (C-3″) 57.65 (C-1″), 76.30 (C-2), 95.35 (C-6), 101.78 (C-10), 104.85 (C-8), 114.98 (C-2′,6′), 127.20 (C-3′,5′), 157.50, 155.53, 154.10, 153.90 (C-5,7,9,4′). Compound **1b**: ^1^H-NMR (400 MHz, DMSO-*d*_6_) δ 1.60 (2H, m, H-5″ and H-4″), 1.88 (4H, m, H-3,4″,5″,6″), 2.09 (2H m, H-3,6″), 2.62 (2H, m, H-4), 2.90 (1H, m, H-3″), 3.00 (1H, m, N-H), 4.32 (1H, m, H-1″), 5.00 (1H, m, H-2), 6.25 (1H, s, H-6), 6.80 (2H, d, H-2′,6′), 7.20 (2H, d, H-3′,5′), ^13^C-NMR (100 MHz, DMSO-*d*_6_) δ 19.58 (C-4), 21.8, 22.75 (C-4″,5″) 28.56 (C-6′’), 28.54 (C-3), 45.40 (C-3″) 57.65 (C-1′″), 76.30 (C-2), 95.35 (C-6), 101.78 (C-10), 104.80 (C-6), 114.98 (C-2′,6′), 127.20 (C-3′,5′), 157.50, 155.53, 154.10, 153.90 (C-5,7,9,4′).

## Supplementary Materials

The following are available online, for related NMR spectra.
