# Supplementary material for: Total Synthesis of Novel Skeleton Flavan-Alkaloids
Source: Molecules. 2020 Sep 30;25(19):4491. doi: 10.3390/molecules25194491 (PMC7582810; doi:10.3390/molecules25194491)
Supplement: Supplementary file 1 [file molecules-25-04491-s001.pdf]

Appendix VI

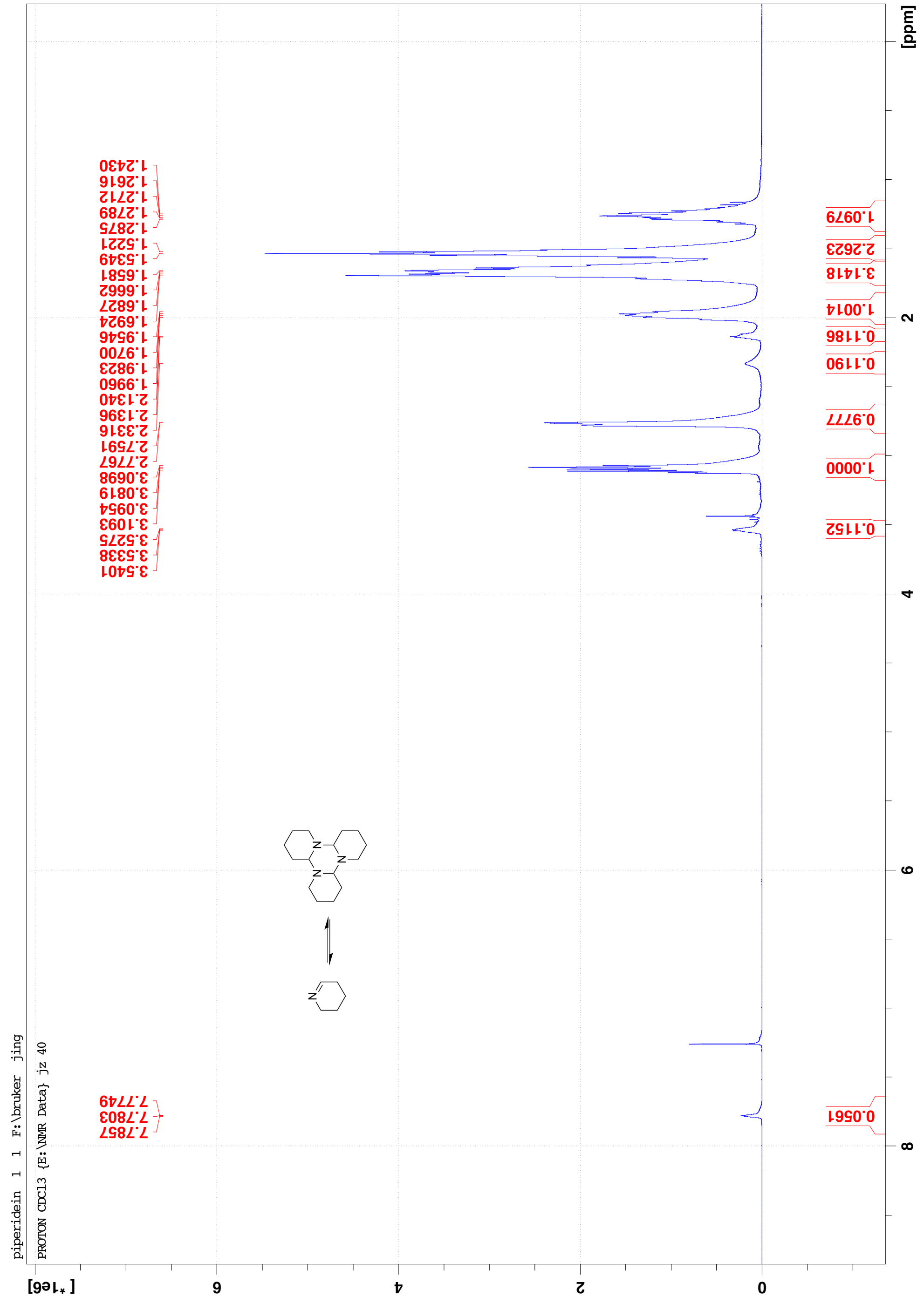

Appendix VI. ^{1}H spectrum of Δ^{1}-piperidein

Appendix VII

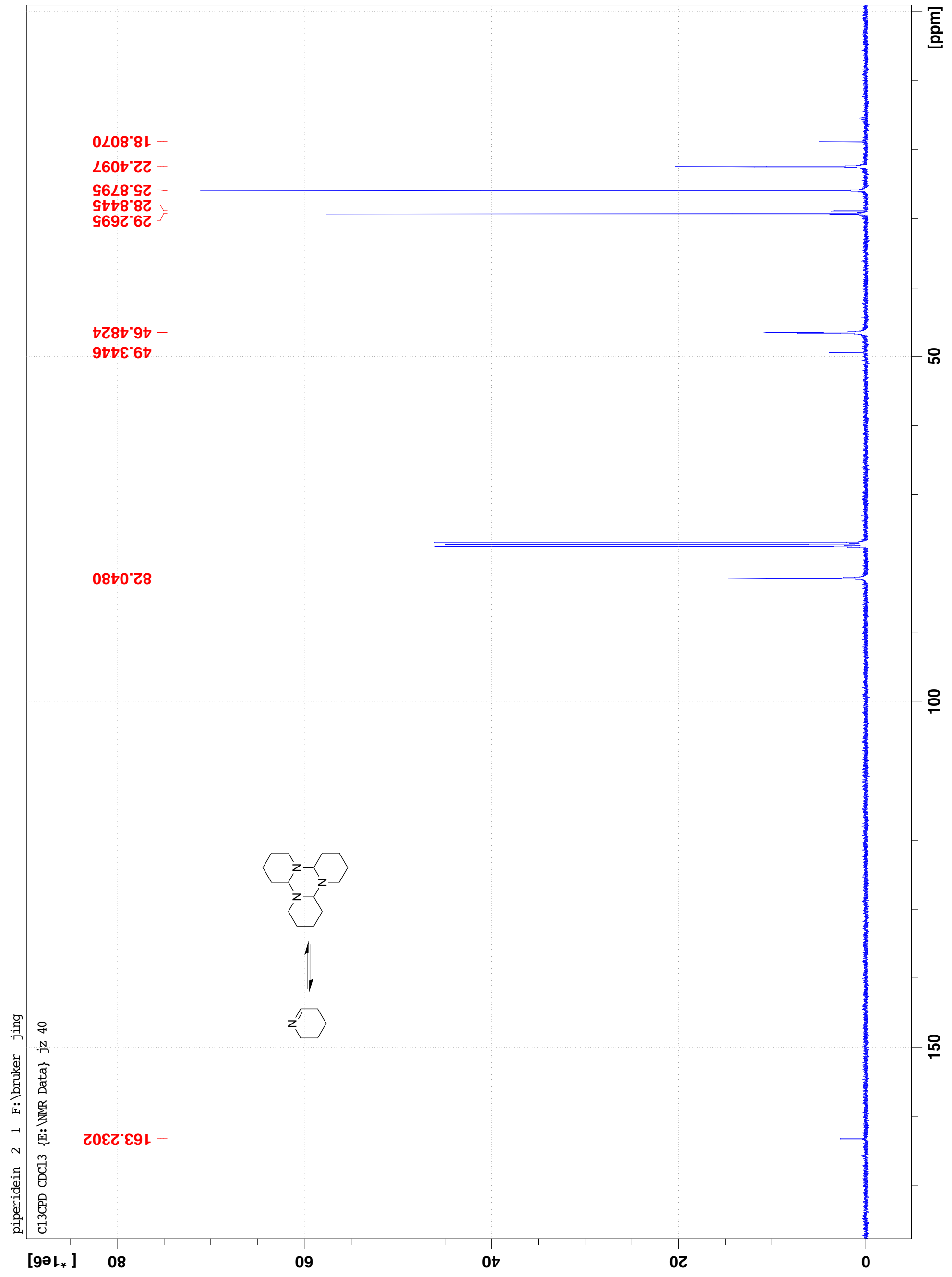

Appendix VII. ^{13}C spectrum of Δ^1-piperidein

Appendix VIII

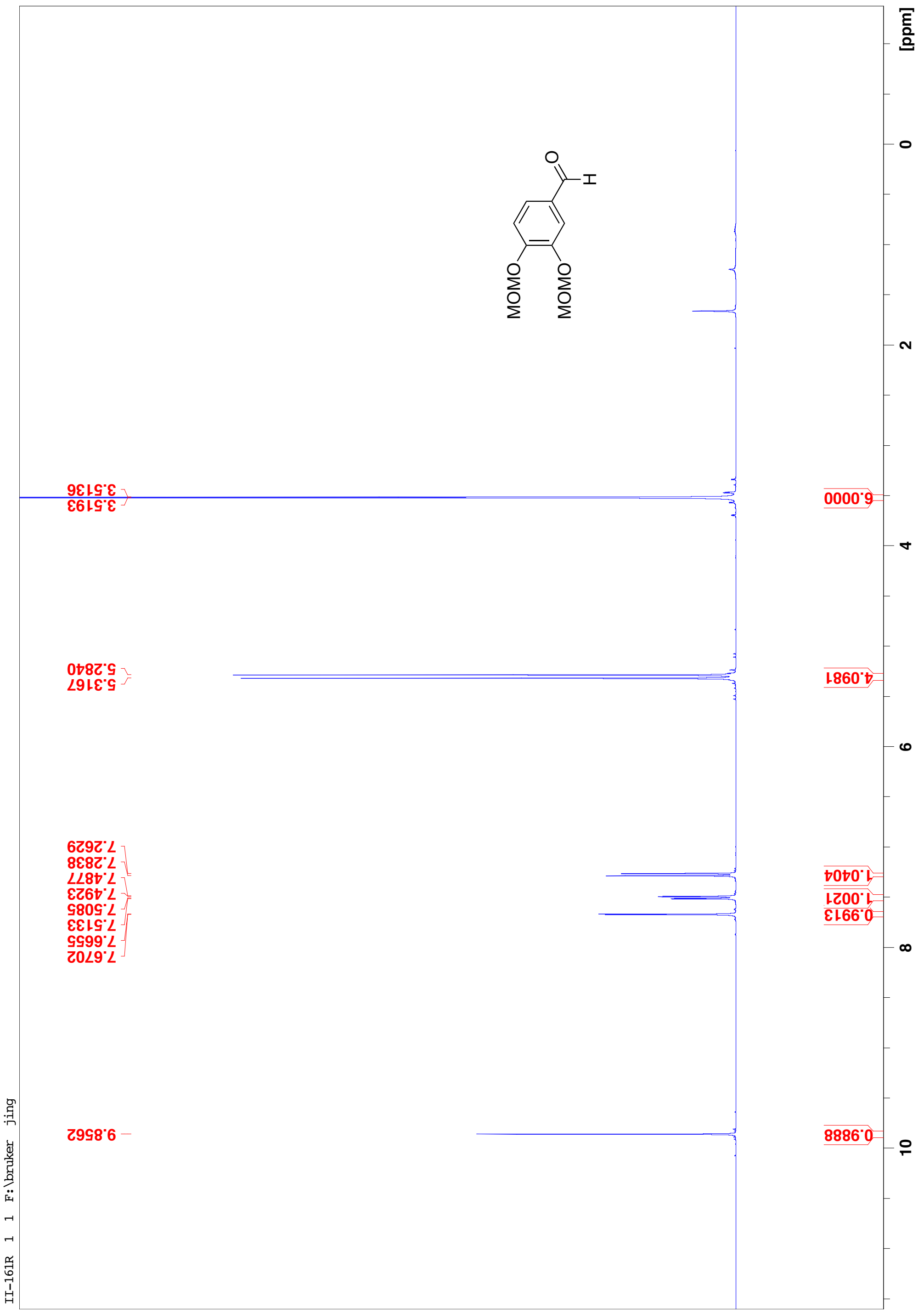

Appendix VIII. ^{1}H spectrum of 3,4-bis(methoxymethoxy)benzaldehyde

Appendix IX

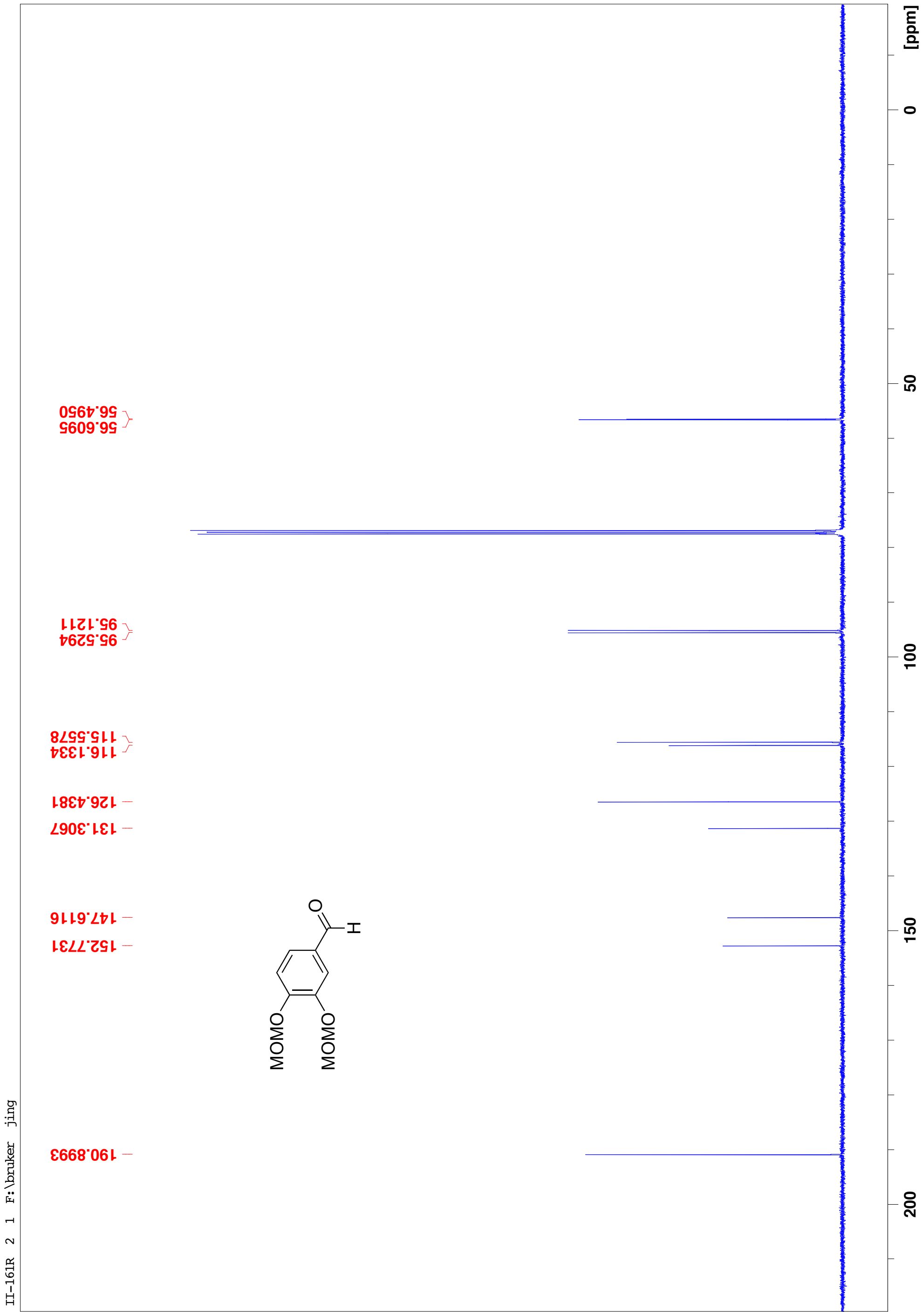

Appendix IX. ^{13}C spectrum of 3,4-bis(methoxymethoxy)benzaldehyde

Appendix X

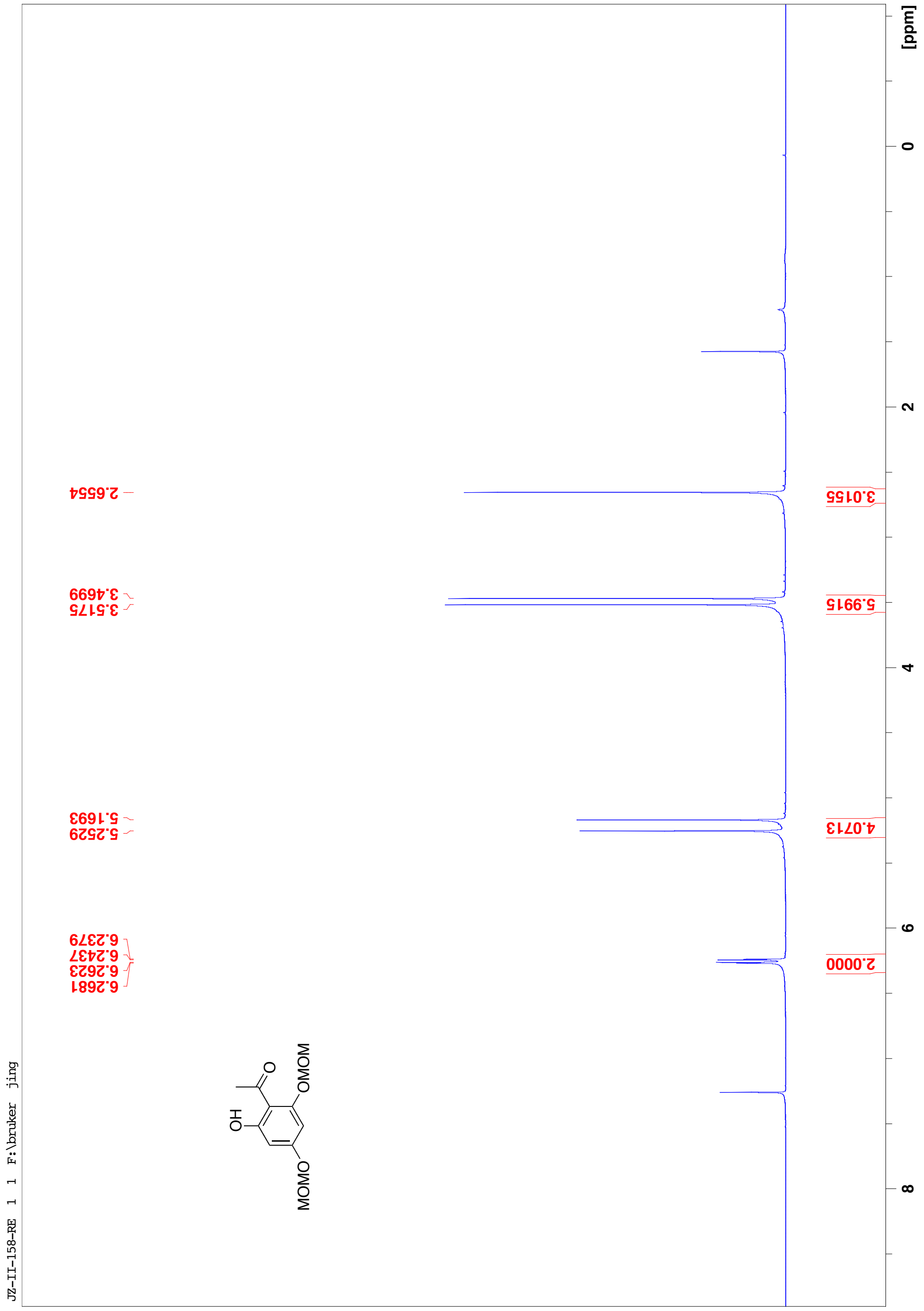

Appendix X. ^{1}H spectrum of 1-(2-hydroxy-4,6-bis(methoxymethoxy)phenyl)ethan-1-

Appendix XI

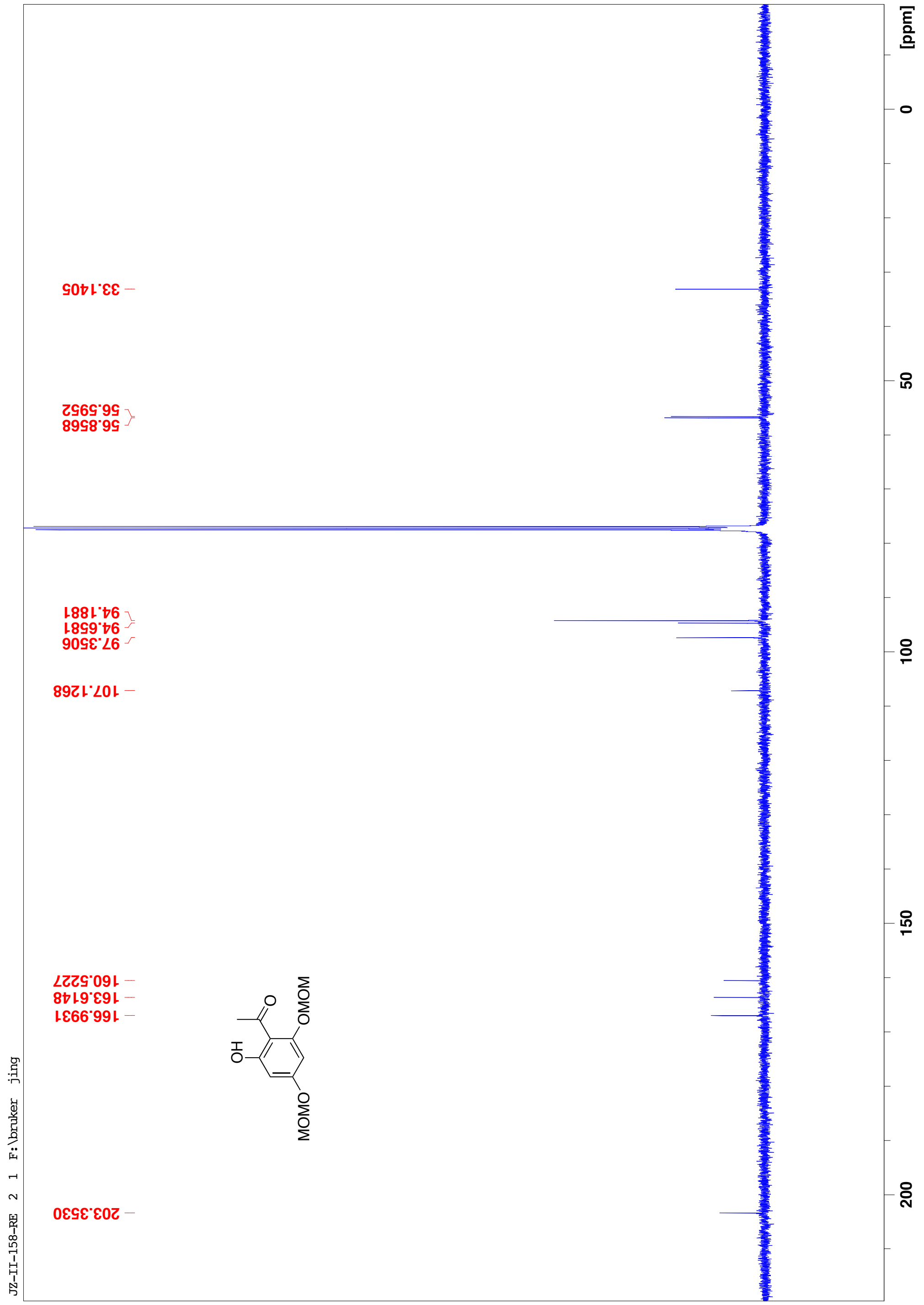

Appendix XI. ^{13}C spectrum of 1-(2-hydroxy-4,6-bis(methoxymethoxy)phenyl)ethan-1-

Appendix XII

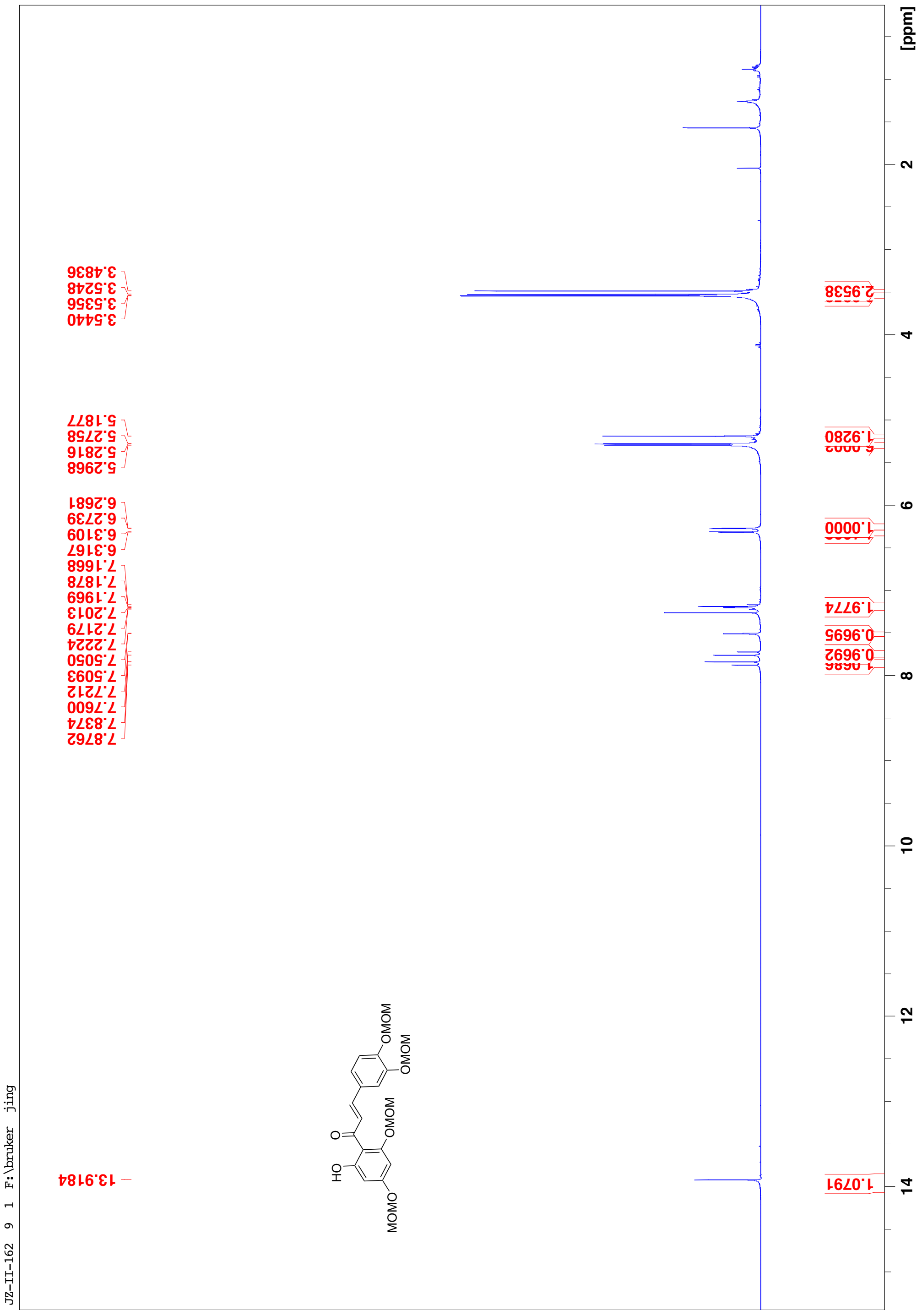

Appendix XII. ^{1}H spectrum of 3-(3,4-bis(methoxymethoxy)phenyl)-1-(2-hydroxy-4,6-bis(methoxymethoxy)phenyl)prop- 2-en-1-one

Appendix XIII

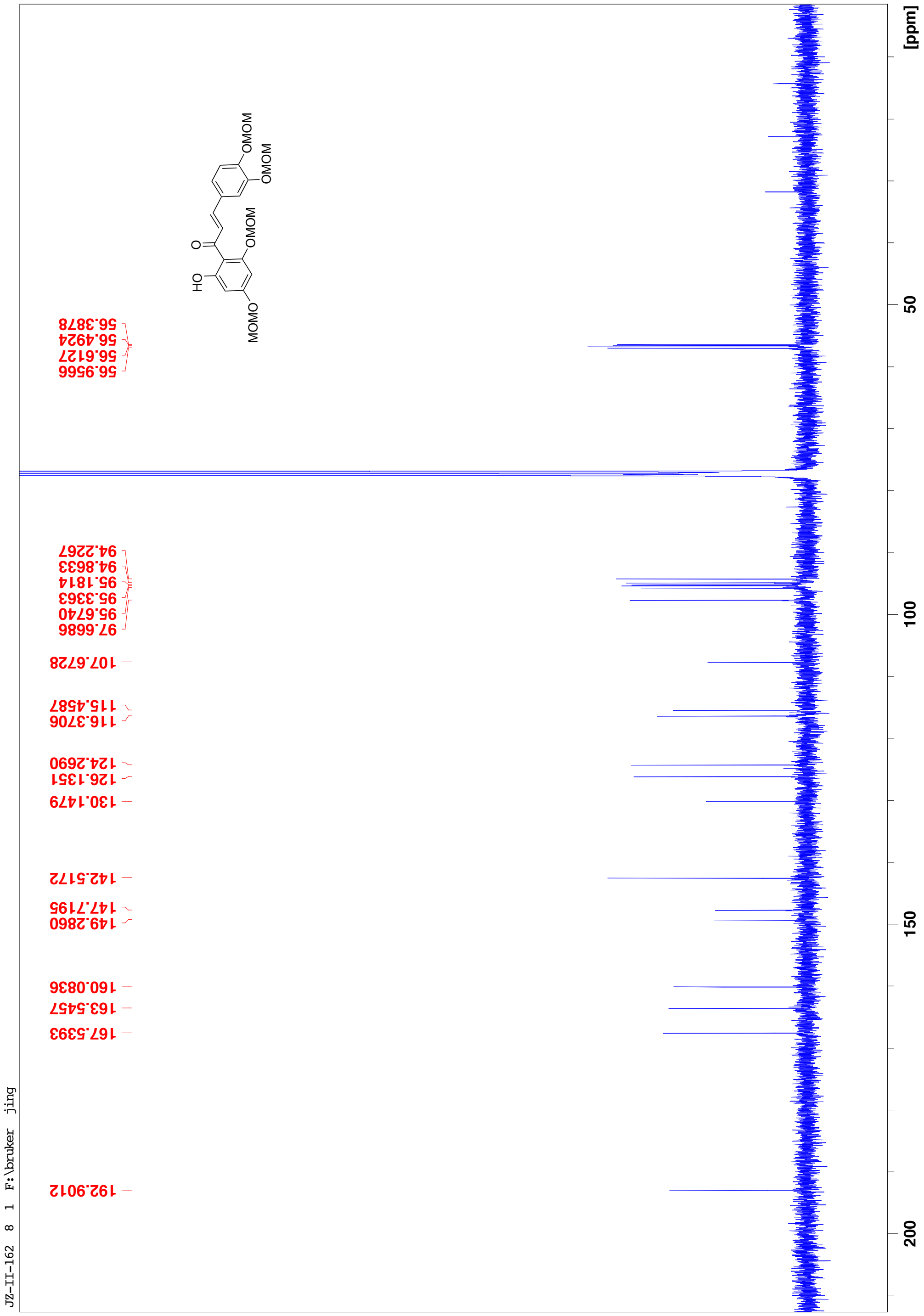

Appendix XIII. ^{13}C spectrum of 3-(3,4-bis(methoxymethoxy)phenyl)-1-(2-hydroxy-4,6-bis(methoxymethoxy)phenyl)prop- 2-en-1-one

Appendix XIV

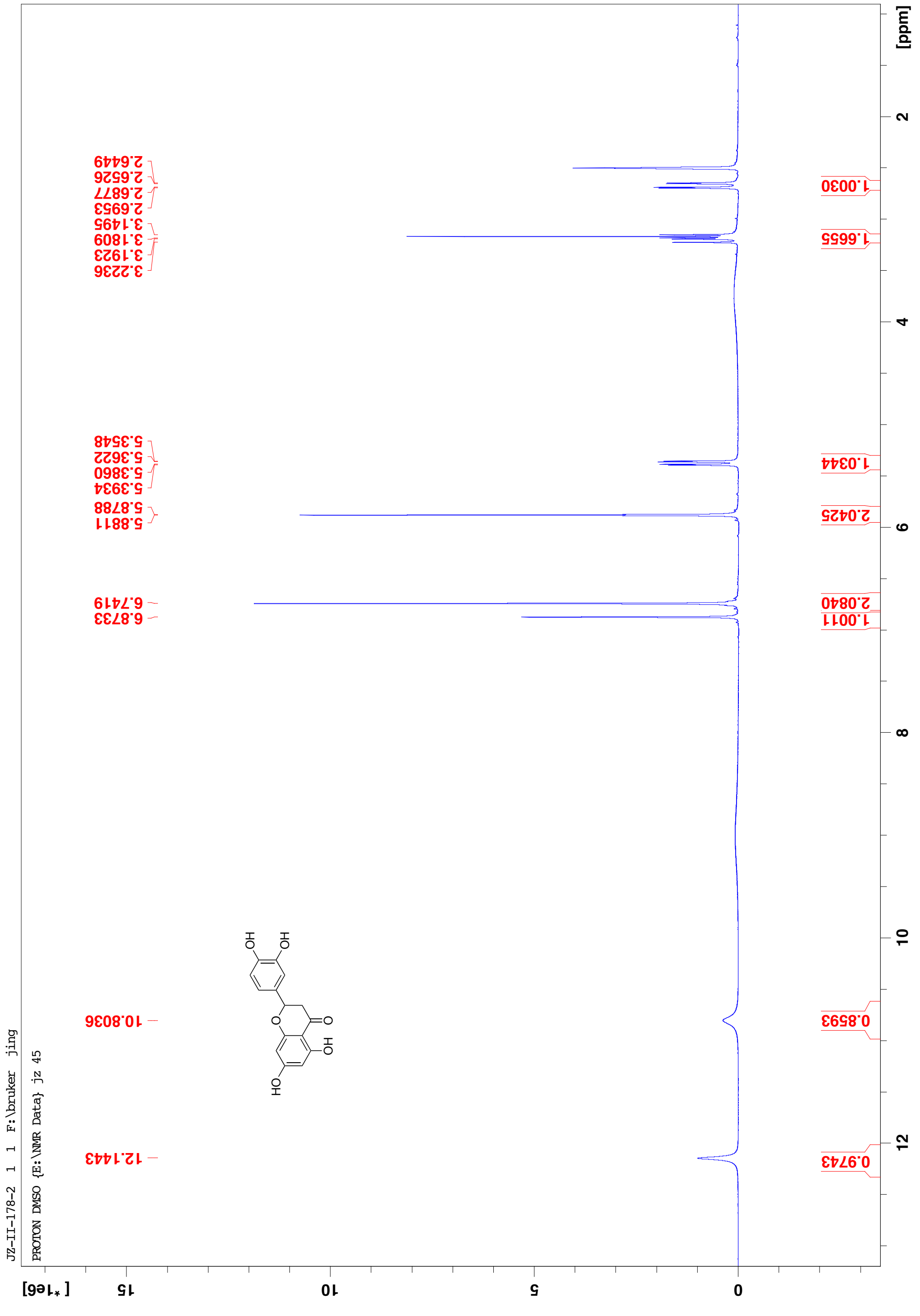

Appendix XIV. ^{1}H spectrum of 2-(3,4-dihydroxyphenyl)-5,7-dihydroxychroman-4-one

Appendix XV

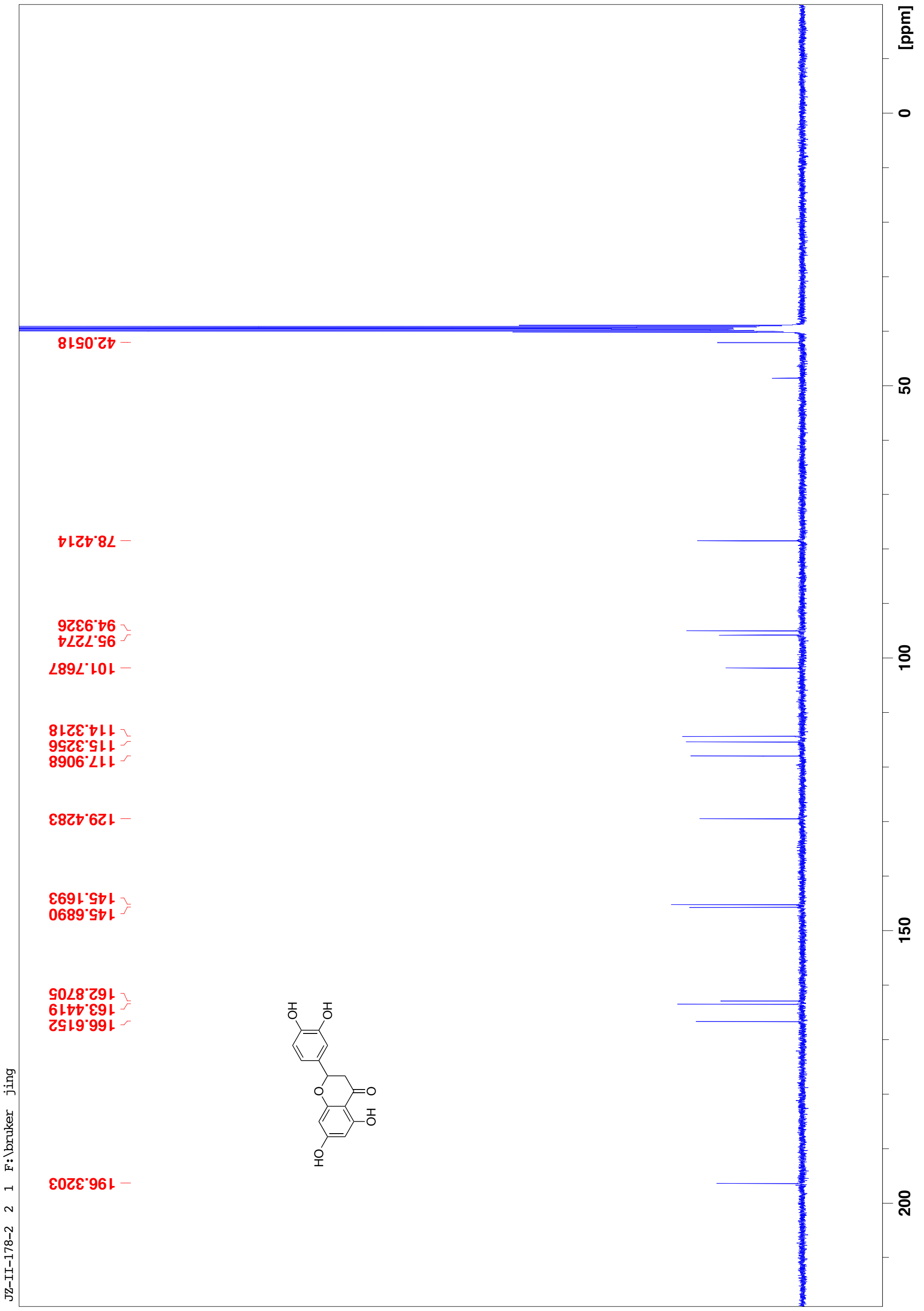

Appendix XV. ^{13}C spectrum of 2-(3,4-dihydroxyphenyl)-5,7-dihydroxychroman-4-one

Appendix XVI

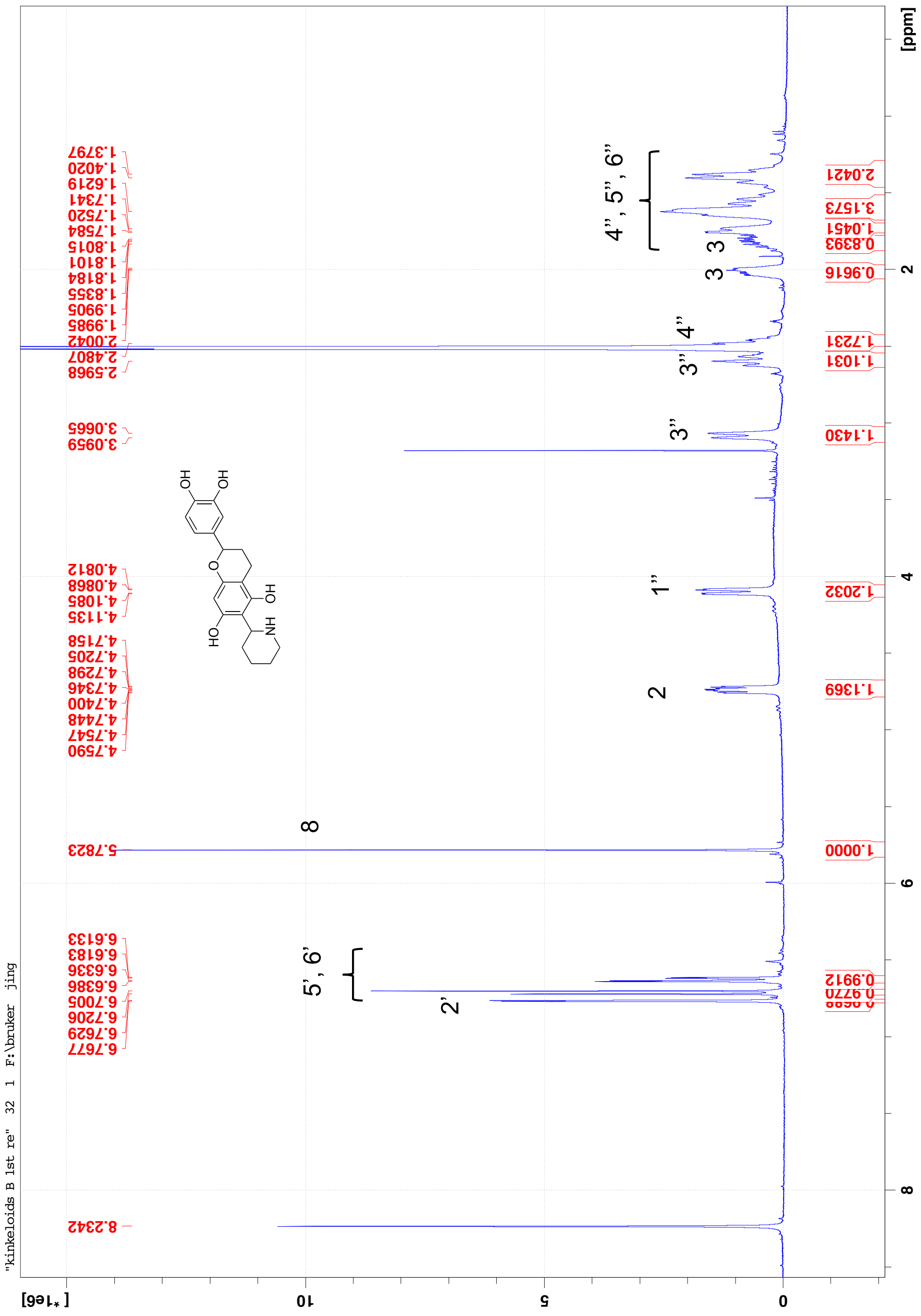

Appendix XVI. ^{1}H spectrum of 6-piperidyl kinkeloids B

Appendix XVII

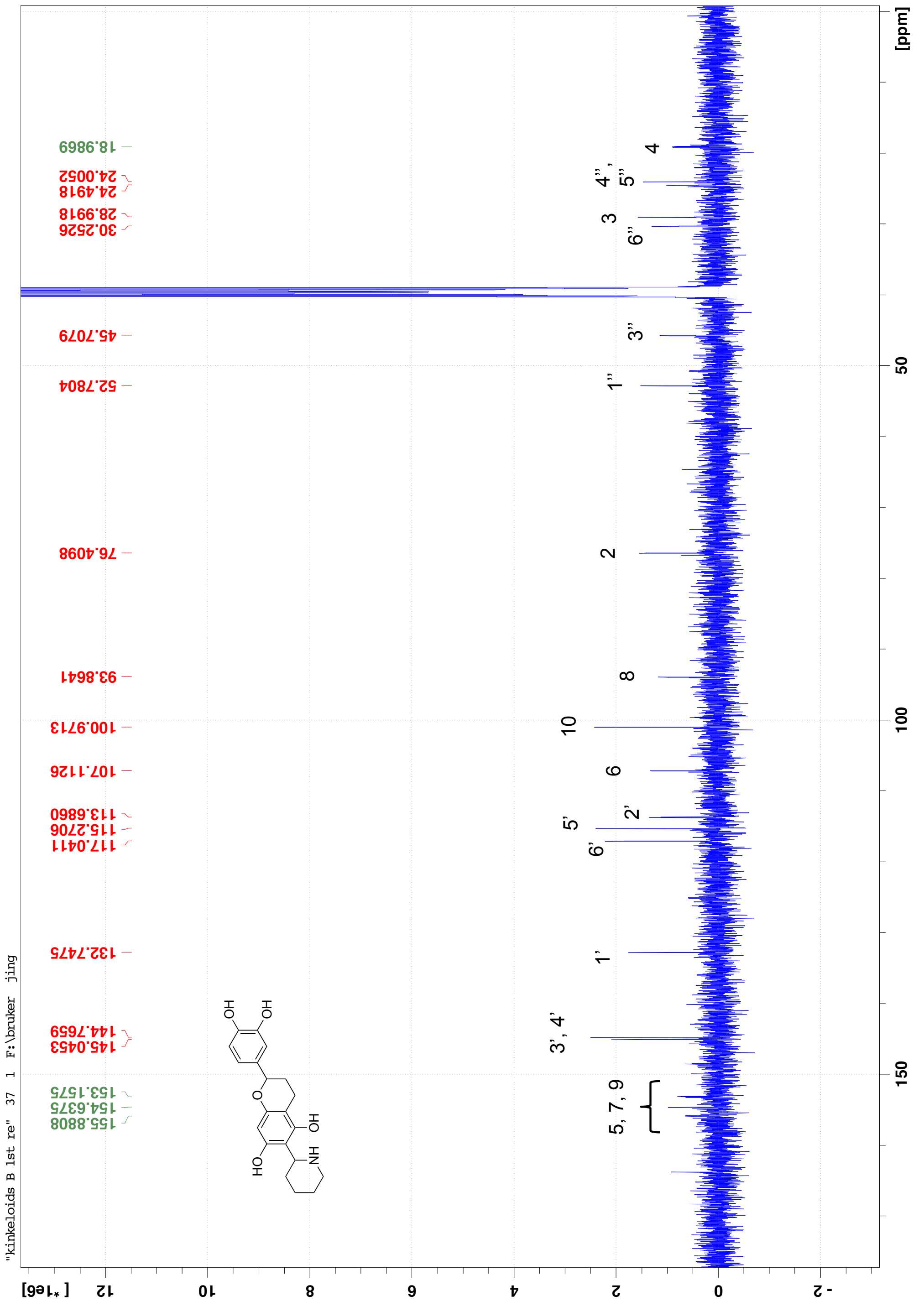

Appendix XVII. ^{13}C spectrum of 6-piperidyl kinkeloids B

Appendix XVIII

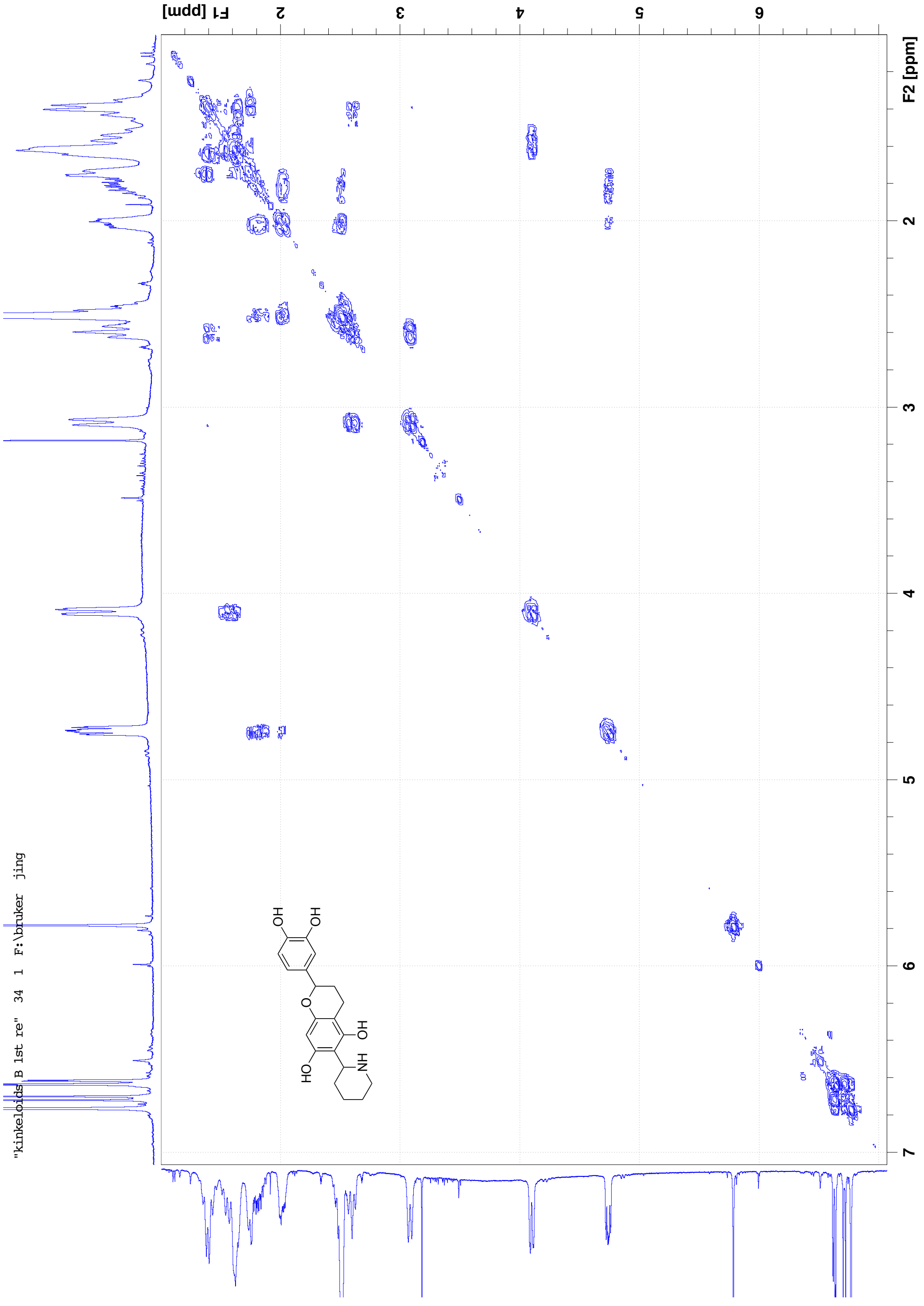

Appendix XVIII. COSY spectrum of 6-piperidyl kinkeloids B

Appendix XIX

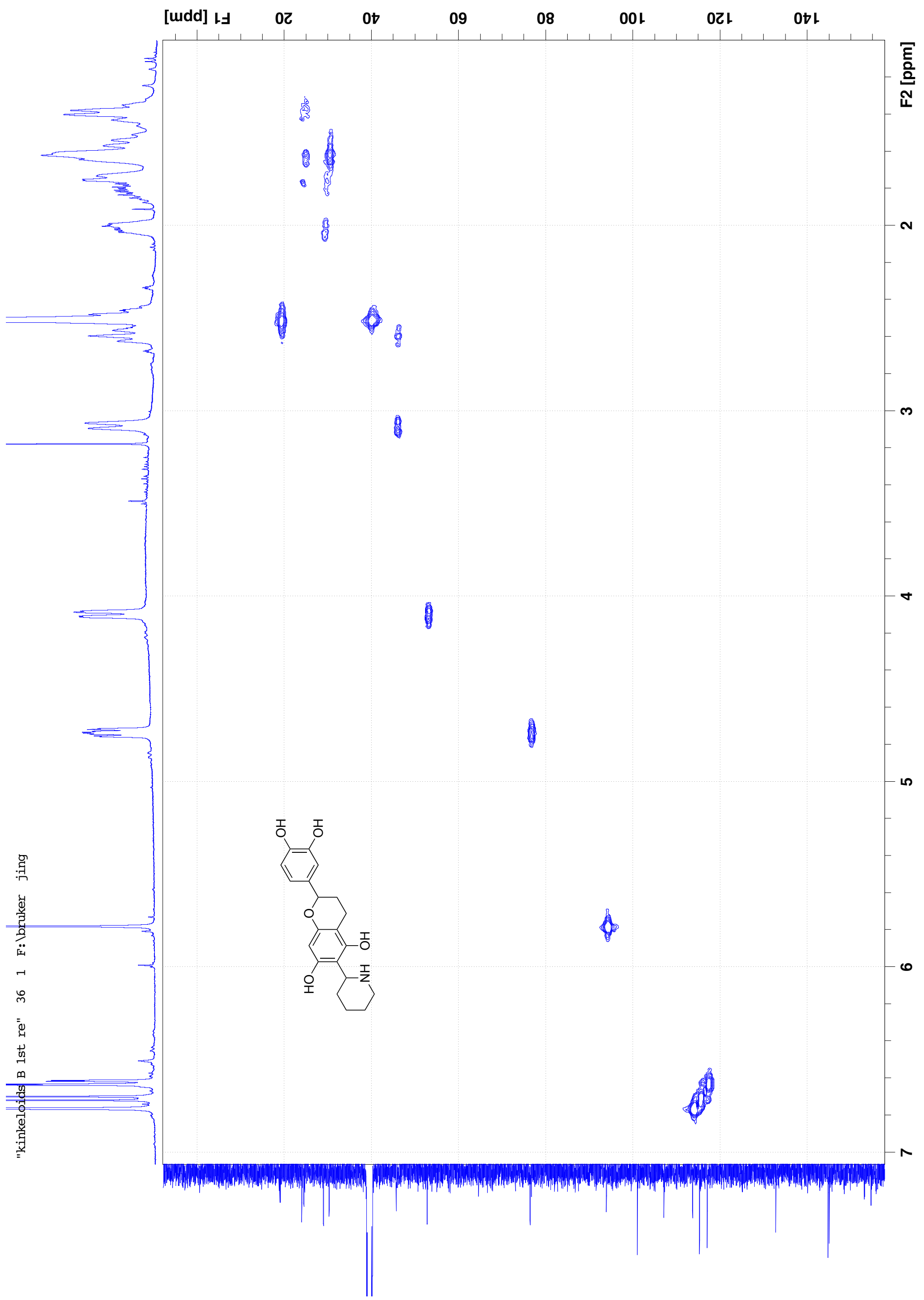

Appendix XIX. HMQC spectrum of 6-piperidyl kinkeloids B

Appendix XX

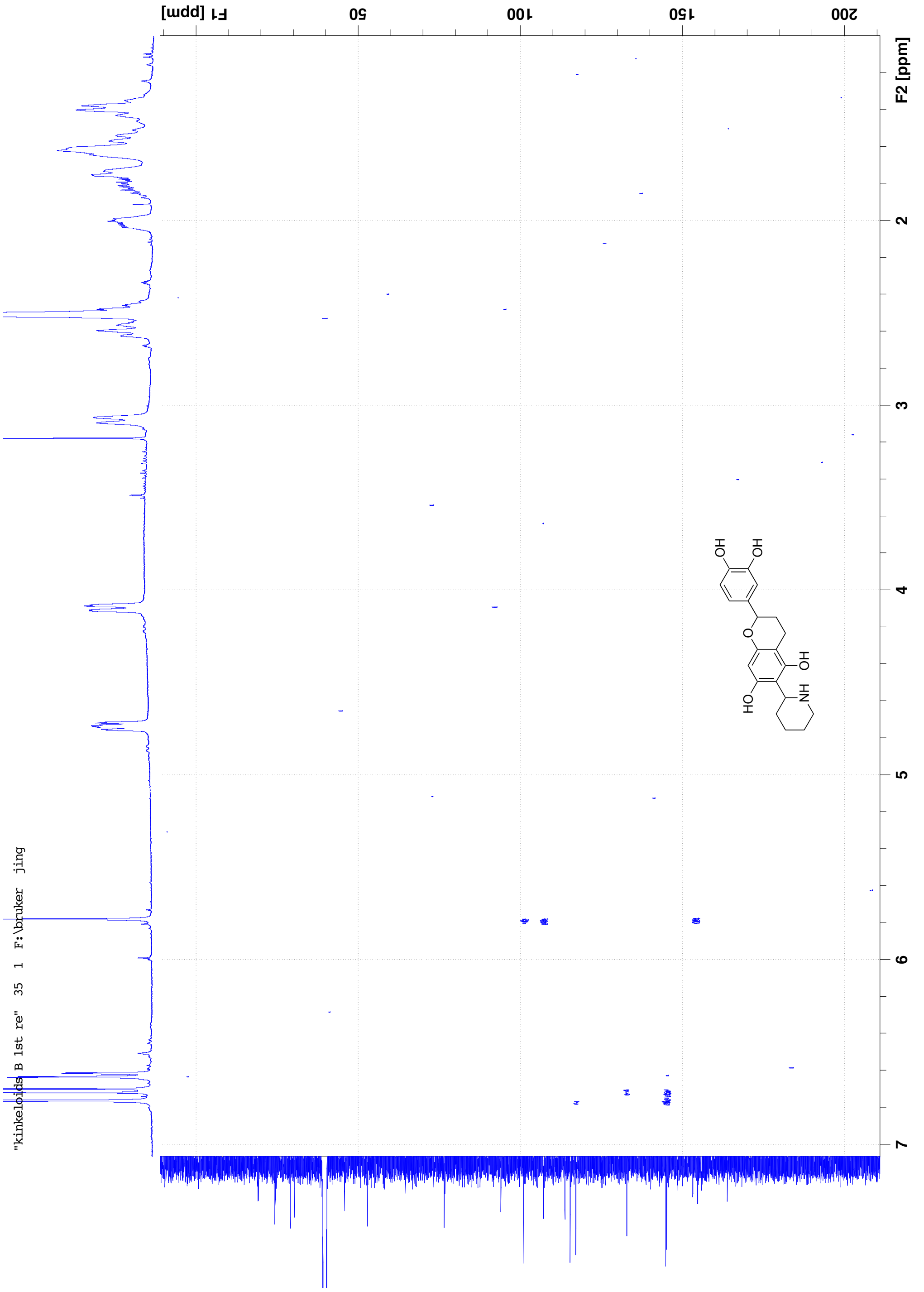

Appendix XX. HMBC spectrum of 6-piperidyl kinkeloids B

Appendix XXI

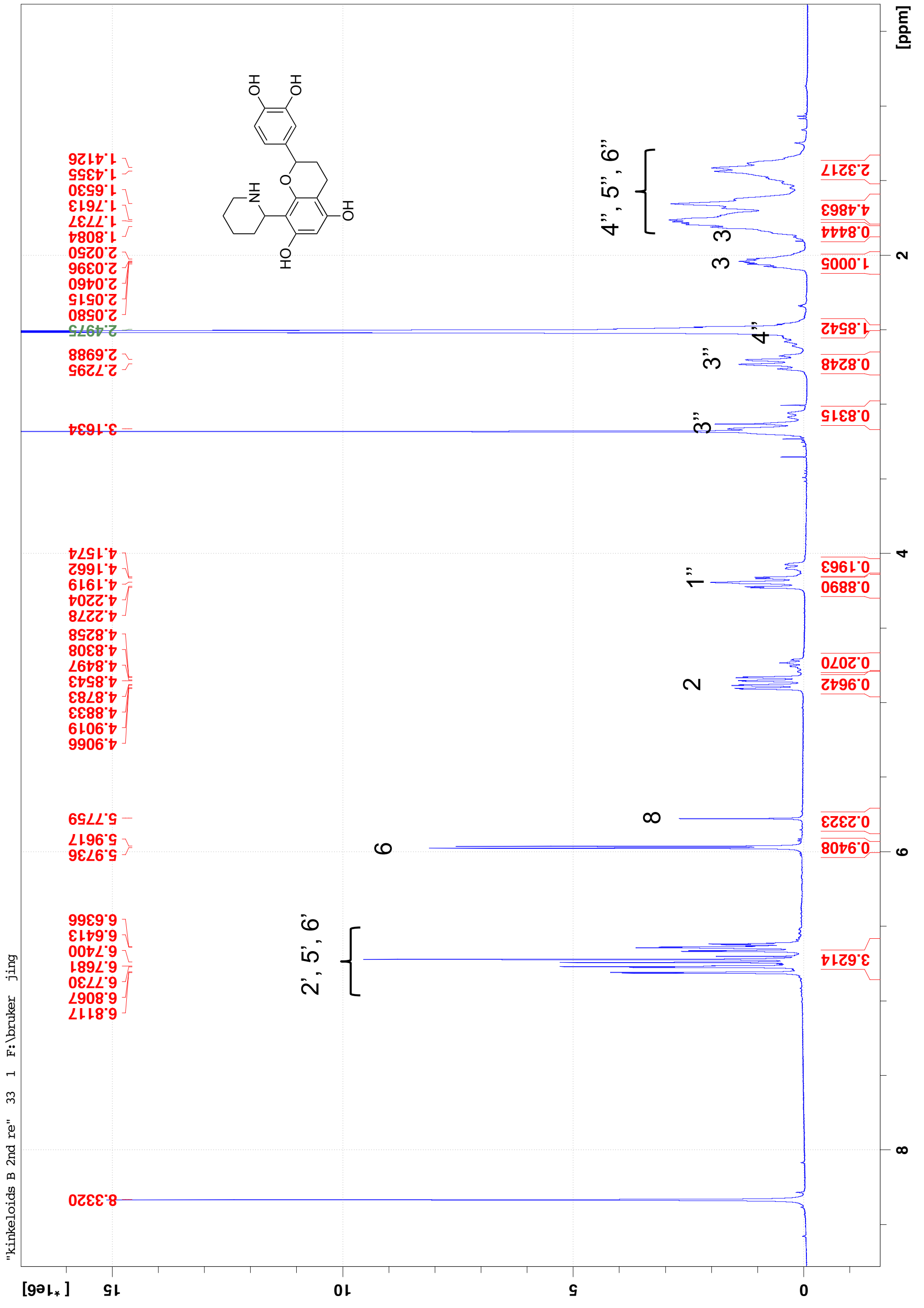

Appendix XXI. ^{1}H spectrum of 8-piperidyl kinkeloids B. (Partially mixed with regioisomers 6-piperidyl kinkeloids B

Appendix XXII

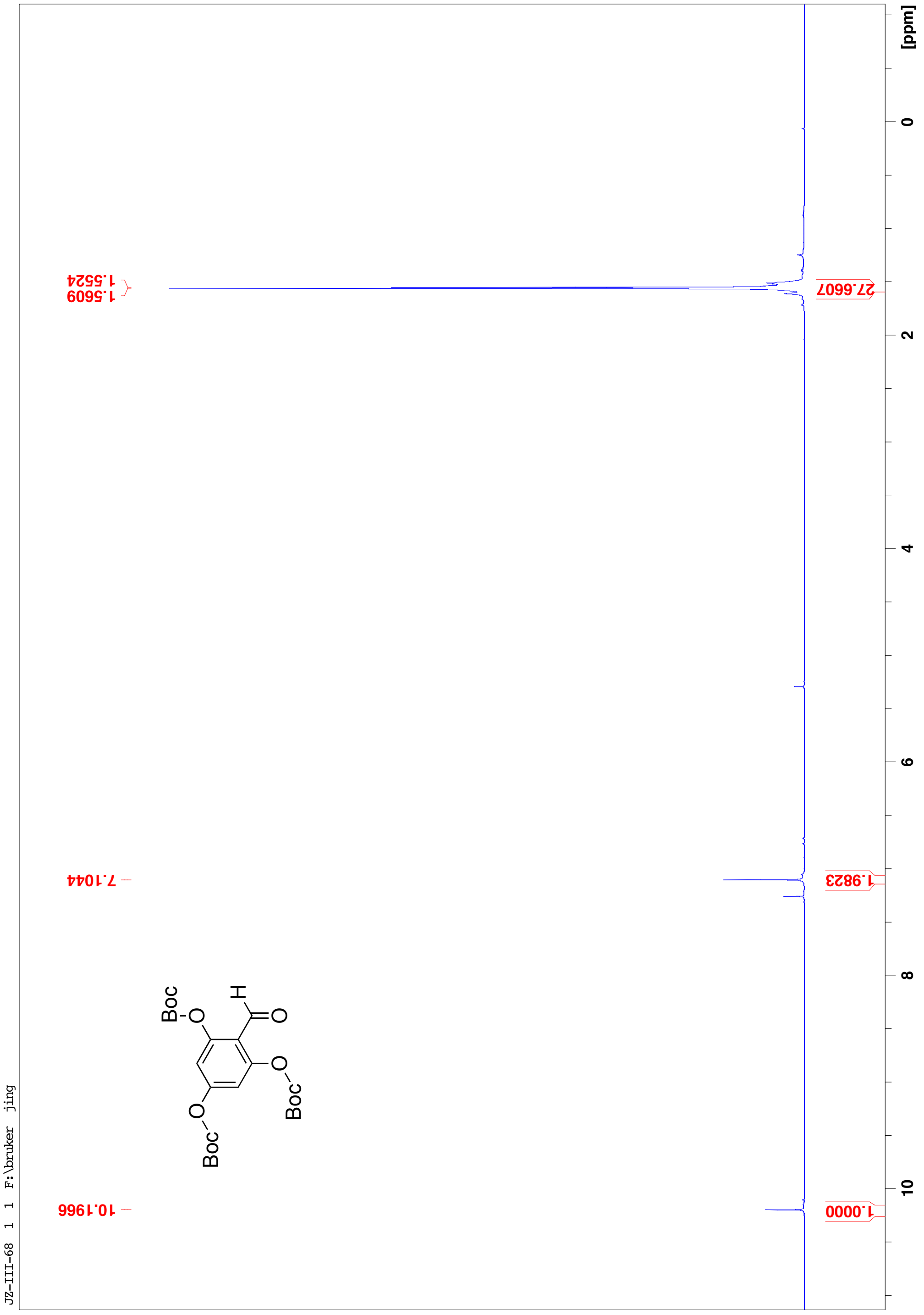

App XXII. ¹H spectrum of tri-tert-butyl (2-formylbenzene-1,3,5-triyl) tricarbonate

Appendix XXIII

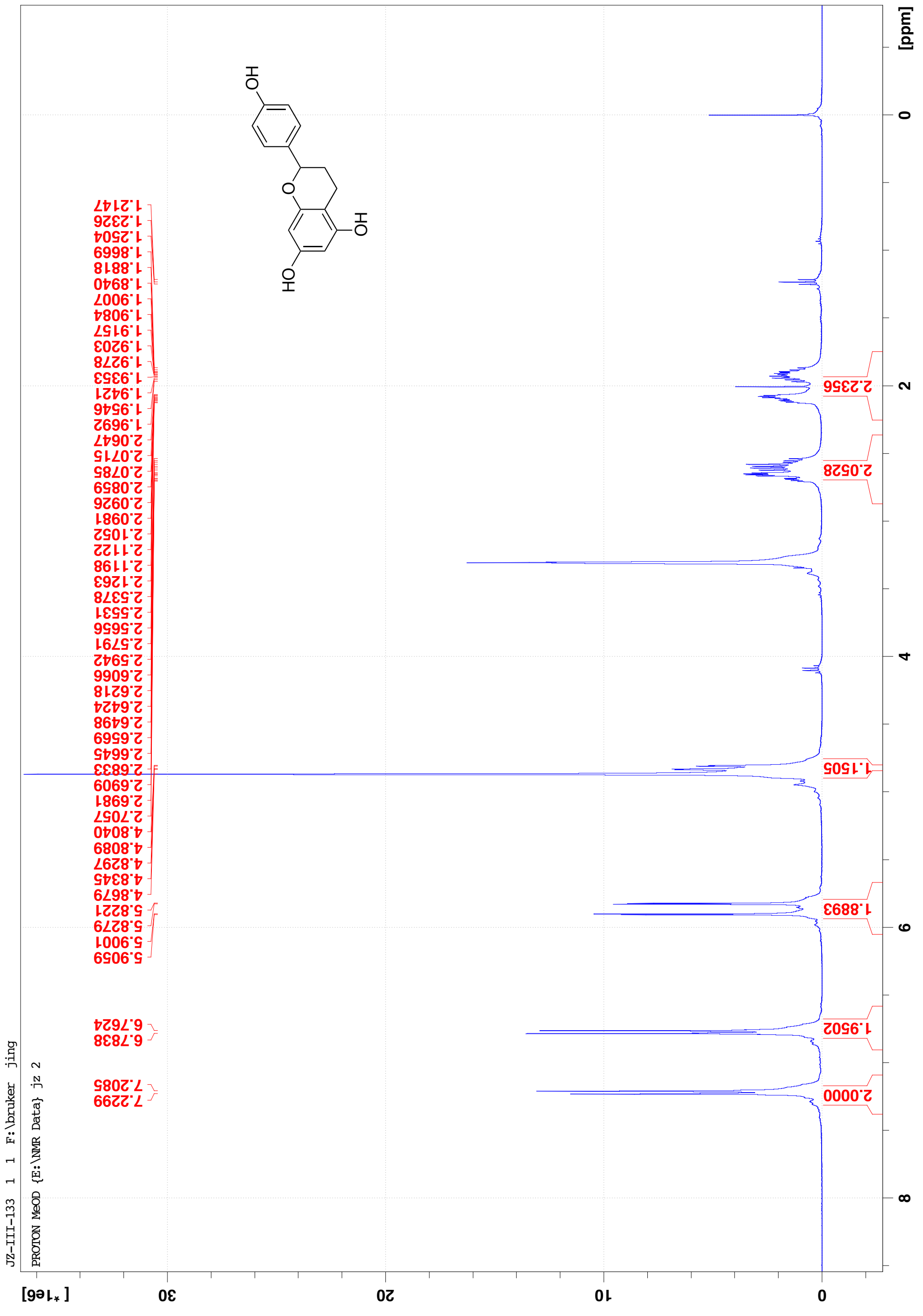

Appendix XXIII. ^{1}H spectrum of 2-(4-hydroxyphenyl)chromane-5,7-diol

Appendix XXIV

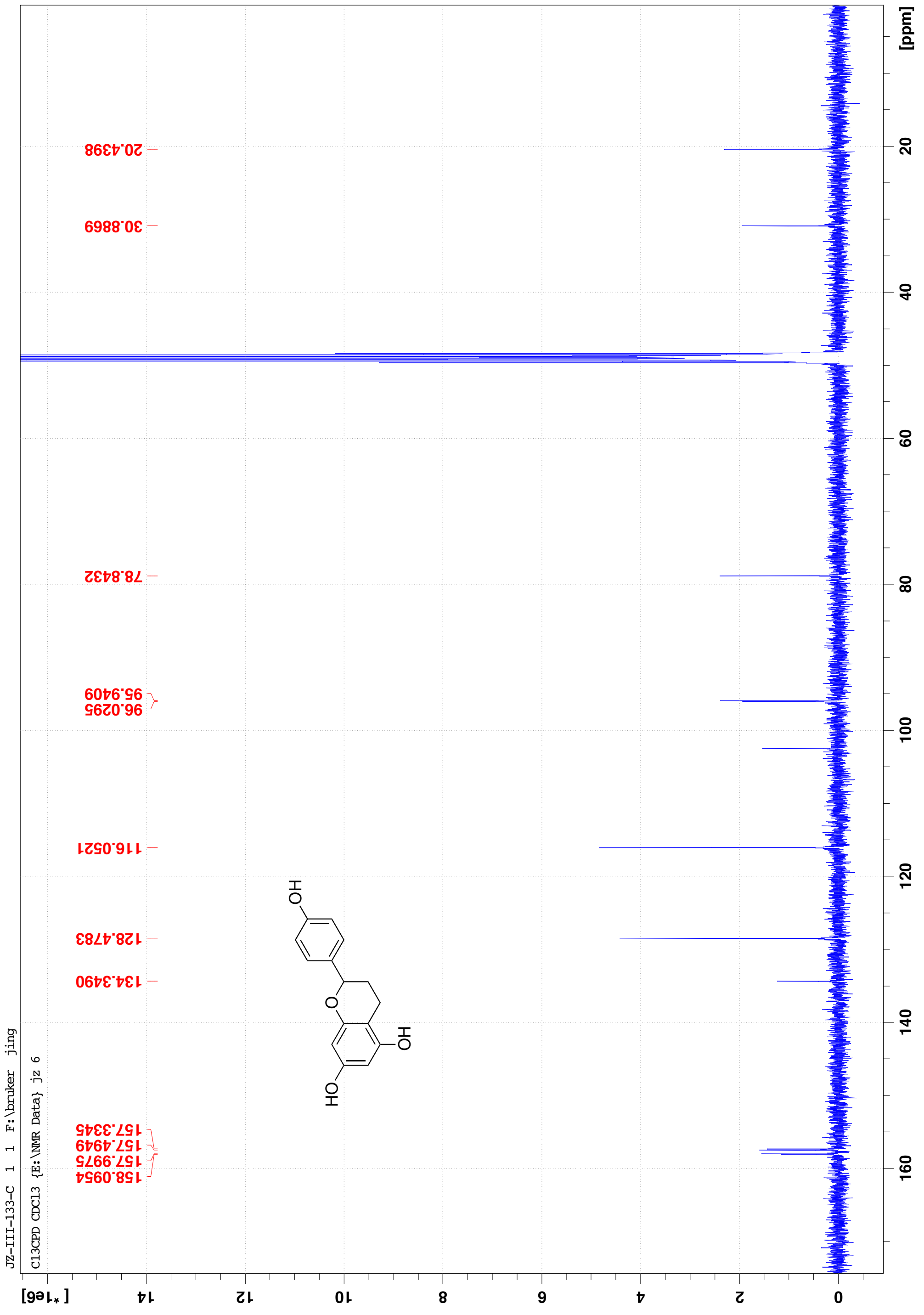

Appendix XXIV. ^{13}C spectrum of 2-(4-hydroxyphenyl)chromane-5,7-diol

Appendix XXV

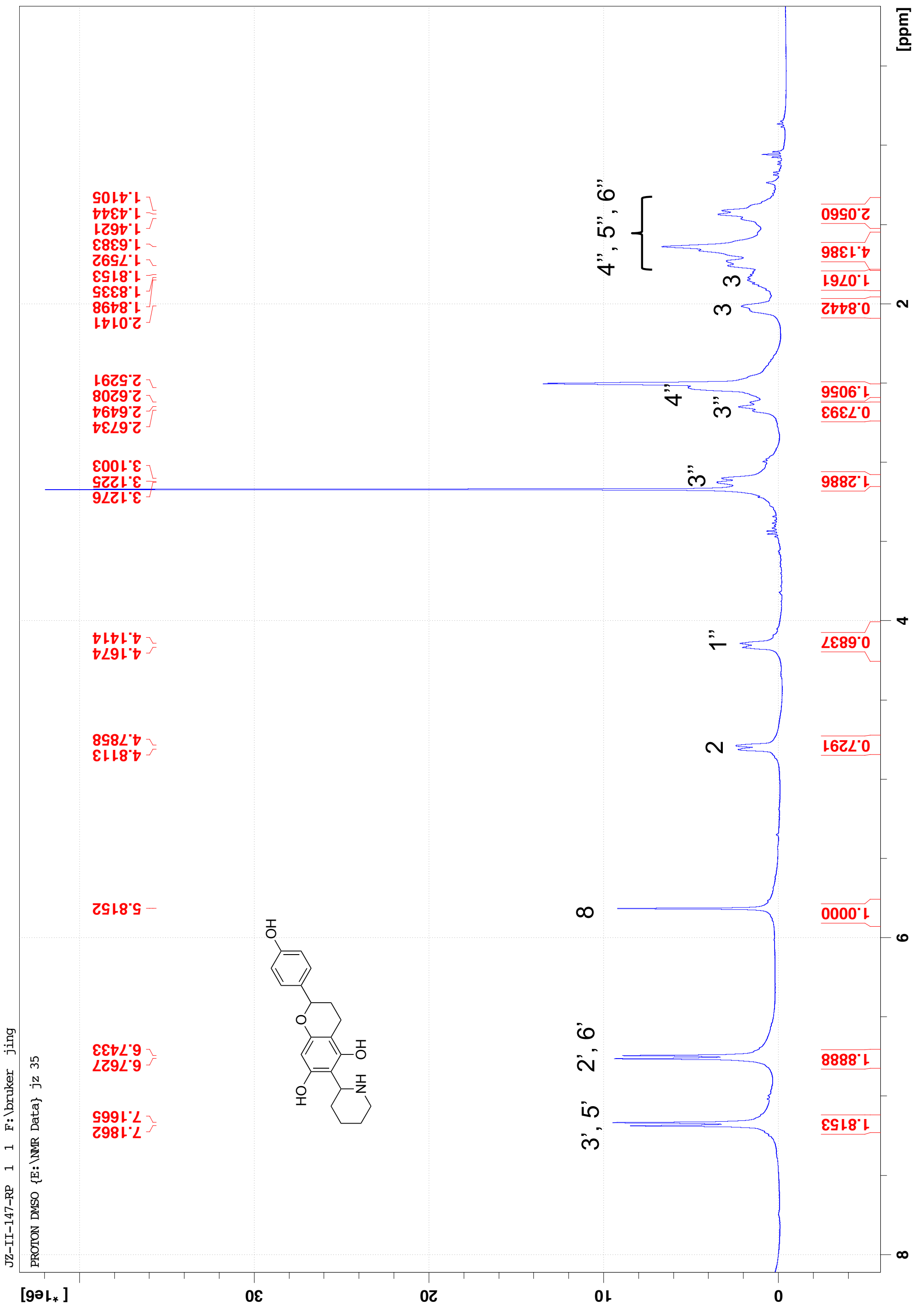

Appendix XXV. ^{1}H spectrum of 6-piperidyl kinkeloids A

Appendix XXVI

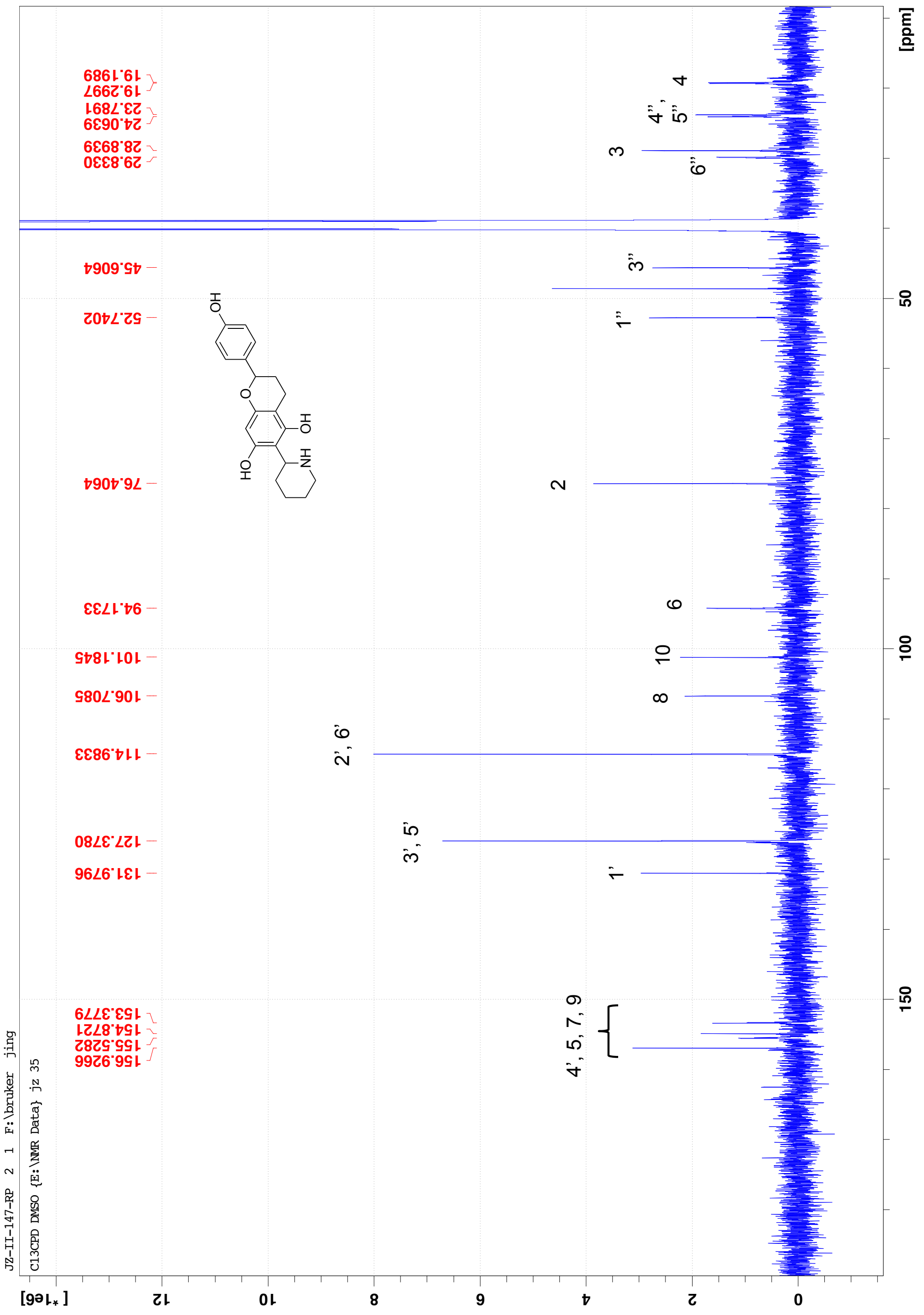

Appendix XXVI. ^{13}C spectrum of 6-piperidyl kinkeloids A

Appendix XXVII

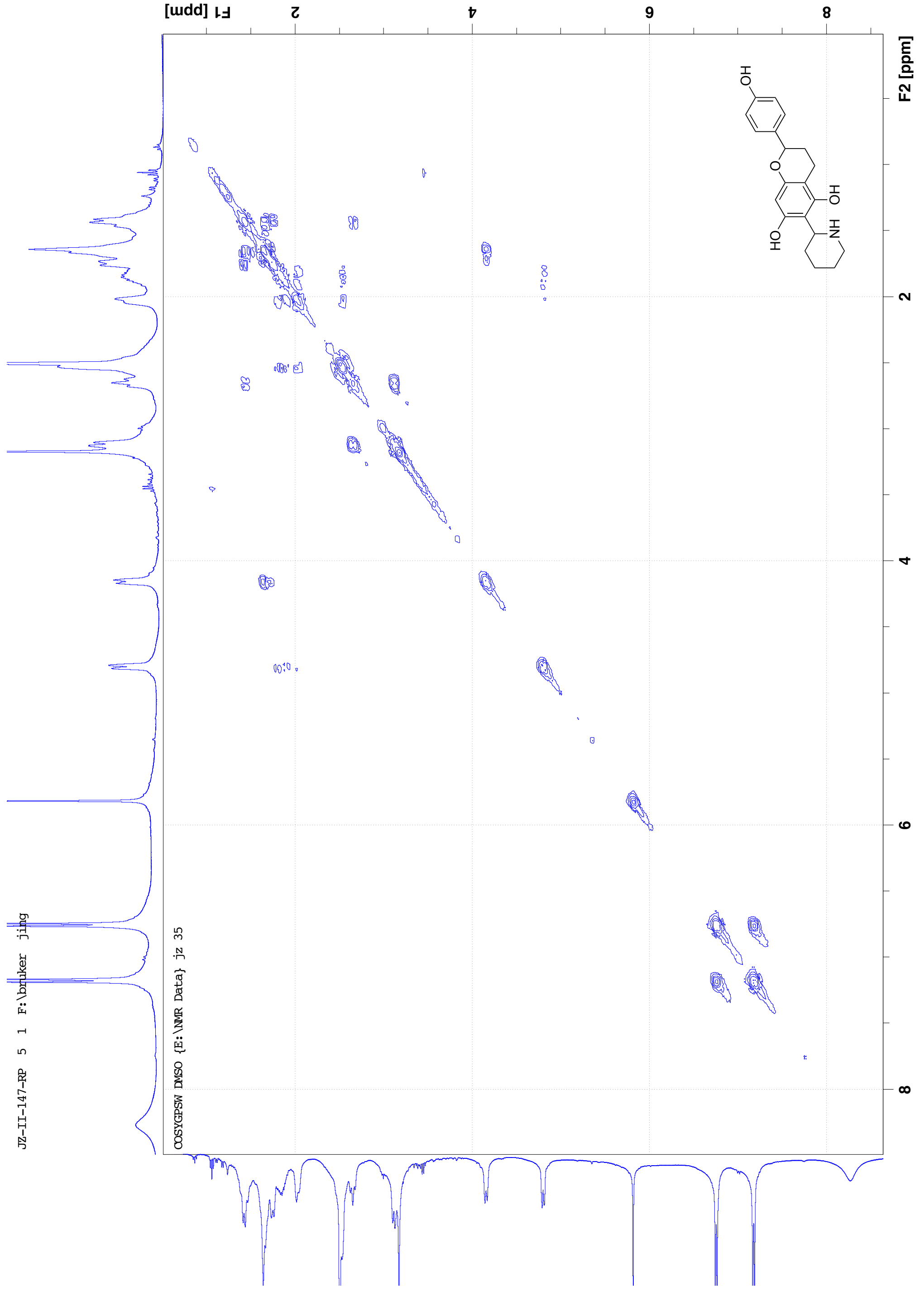

Appendix XXVII. COSY spectrum of 6-piperidyl kinkeloids A

Appendix XXVIII

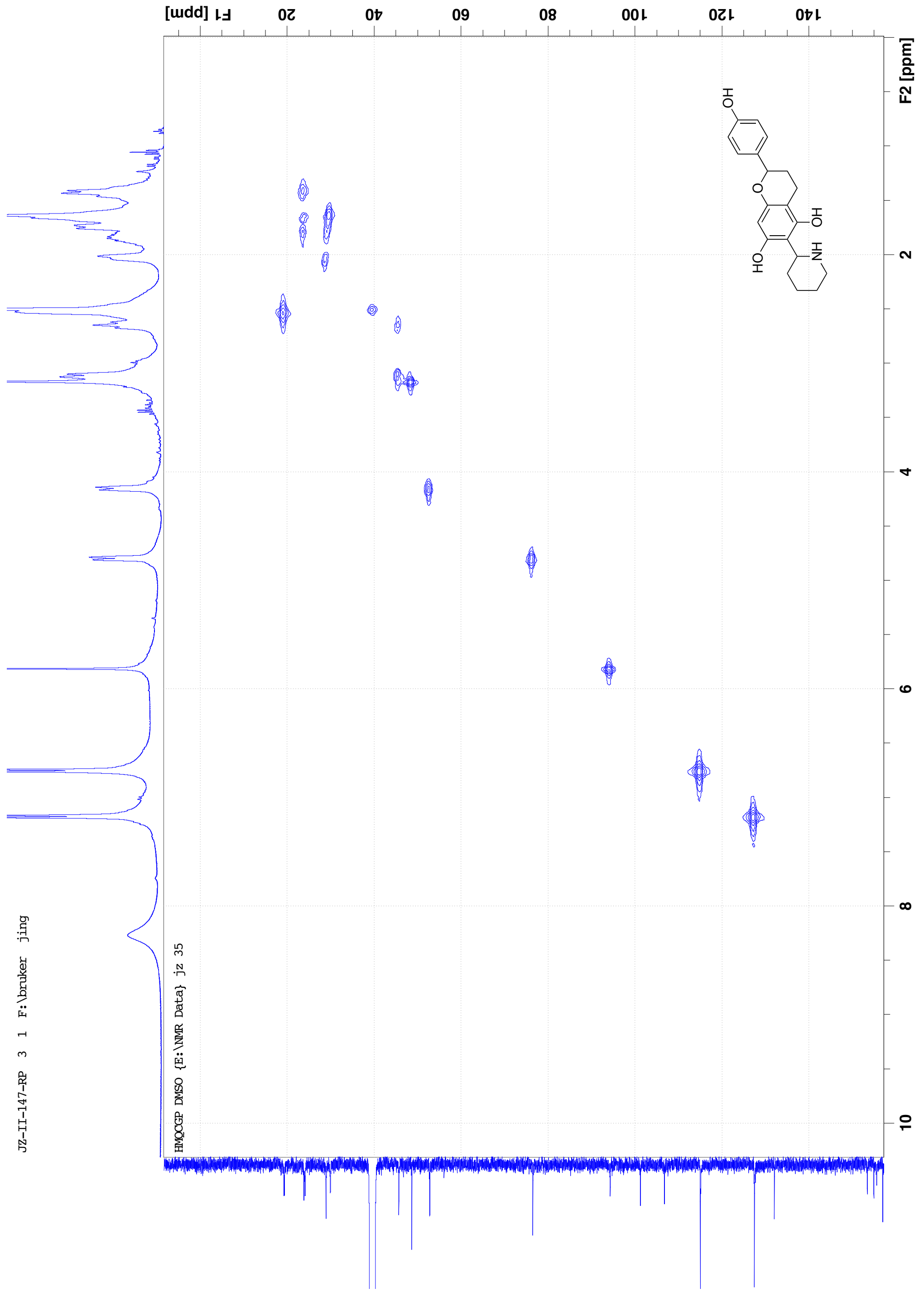

Appendix XXVIII. HMQC spectrum of 6-piperidyl kinkeloids A

Appendix XXIX

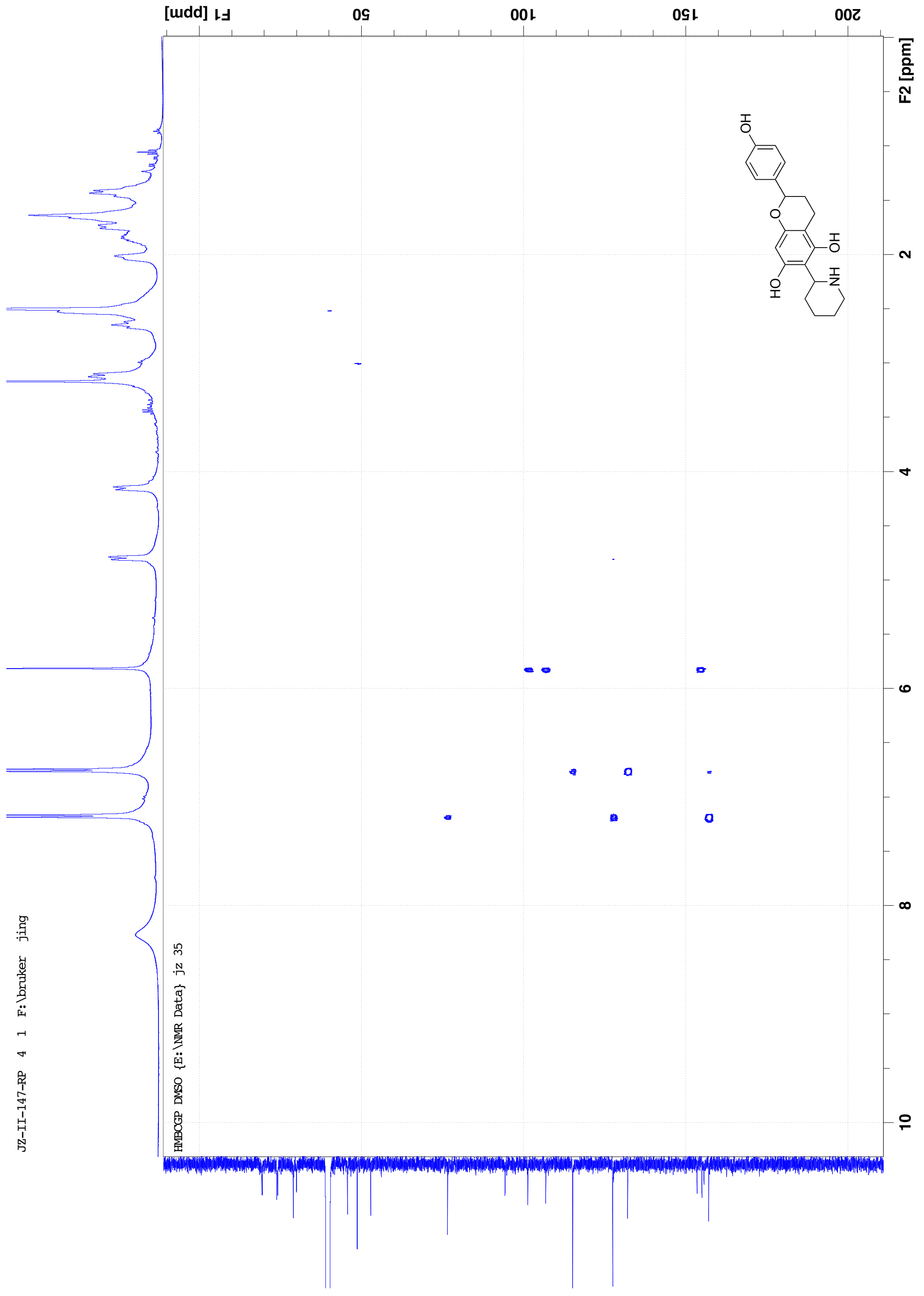

Appendix XXIX. HMBC spectrum of 6-piperidyl kinkeloids A

Appendix XXX

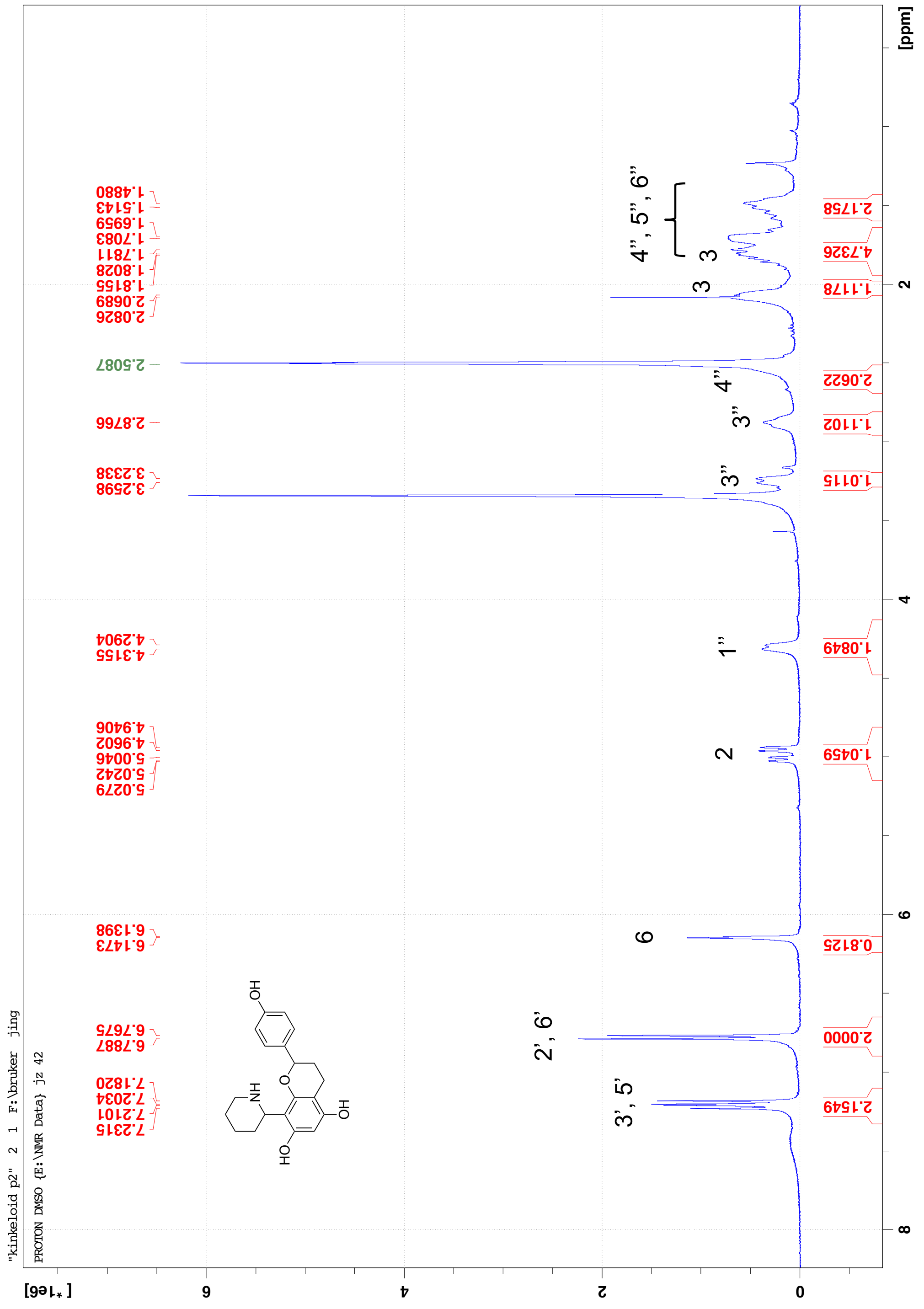

Appendix XXX. ^{1}H spectrum of 8-piperidyl kinkeloids A
